# Transcriptional changes in the rat brain induced by repetitive transcranial magnetic stimulation

**DOI:** 10.3389/fnhum.2023.1215291

**Published:** 2023-11-13

**Authors:** Marina Weiler, Kevin C. Stieger, Kavisha Shroff, Jessie P. Klein, William H. Wood, Yongqing Zhang, Prabha Chandrasekaran, Elin Lehrmann, Simonetta Camandola, Jeffrey M. Long, Mark P. Mattson, Kevin G. Becker, Peter R. Rapp

**Affiliations:** ^1^Laboratory of Behavioral Neuroscience, National Institute on Aging, National Institutes of Health, Baltimore, MD, United States; ^2^Laboratory of Genetics and Genomics, National Institute on Aging, National Institutes of Health, Baltimore, MD, United States; ^3^Laboratory of Clinical Investigation, National Institute on Aging, National Institutes of Health, Baltimore, MD, United States; ^4^Laboratory of Neurosciences, National Institute on Aging, National Institutes of Health, Baltimore, MD, United States

**Keywords:** non-invasive brain stimulation, microarray, gene expression, aging, genomic

## Abstract

**Introduction:**

Transcranial Magnetic Stimulation (TMS) is a noninvasive technique that uses pulsed magnetic fields to affect the physiology of the brain and central nervous system. Repetitive TMS (rTMS) has been used to study and treat several neurological conditions, but its complex molecular basis is largely unexplored.

**Methods:**

Utilizing three experimental rat models (*in vitro*, *ex vivo*, and *in vivo*) and employing genome-wide microarray analysis, our study reveals the extensive impact of rTMS treatment on gene expression patterns.

**Results:**

These effects are observed across various stimulation protocols, in diverse tissues, and are influenced by time and age. Notably, rTMS-induced alterations in gene expression span a wide range of biological pathways, such as glutamatergic, GABAergic, and anti-inflammatory pathways, ion channels, myelination, mitochondrial energetics, multiple neuron-and synapse-specific genes.

**Discussion:**

This comprehensive transcriptional analysis induced by rTMS stimulation serves as a foundational characterization for subsequent experimental investigations and the exploration of potential clinical applications.

## Introduction

Transcranial Magnetic Stimulation (TMS) is a noninvasive technique that uses pulsed magnetic fields to affect the physiology of the brain and the central nervous system (Wagner et al., 2007). Repetitive TMS (rTMS) has been shown to alter higher-order biological processes including neuronal plasticity (Ferreri and Rossini, 2013), cortical excitability (Cavaleri et al., 2017), and cognition (Cheng et al., 2017). rTMS has been used in the study and treatment of neurological conditions including stroke (Smith and Stinear, 2016; McDonnell and Stinear, 2017), epilepsy (Chen et al., 2016), dystonia (Lozeron et al., 2016), schizophrenia (Kaskie and Ferrarelli, 2017), multiple sclerosis (Simpson and Macdonell, 2015), Parkinson’s (Chung and Mak, 2016) and Alzheimer’s disease (Nardone et al., 2014; Weiler et al., 2020), as well as other neurological and non-neurological disorders (Weiler et al., 2021), and was approved for clinical use for treatment-resistant depression by the FDA in 2008.

Remarkably, given its extensive clinical application, the complex molecular basis of rTMS remains largely unexplored. Only a limited number of transcripts and proteins have been previously reported altered following rTMS (Lee et al., 2014; Wang et al., 2014; Cirillo et al., 2017) including *Fos* (Legrand et al., 2018), *Caspase-3* (Grehl et al., 2015), *Gfap* (Grehl et al., 2015), the *MAPK* signaling pathway (Cui et al., 2019), and the miR-409-3p/CTR3/AMPK/Sirt1 axis (Wu et al., 2022). However, a systematic genome-wide transcriptional analysis has not been performed to date (Ikeda et al., 2017, 2018), and the underlying molecular basis of the clinical effects of rTMS treatment remains unknown.

Here, using three experimental models in rats, *in vitro, ex vivo*, and *in vivo*, using genome-wide microarray analysis, we show that rTMS treatment results in broad-based alterations in gene expression patterns using different stimulation protocols, in different tissues, over time, and with age. Gene expression was altered due to rTMS stimulation in numerous biological pathways including glutamatergic and GABAergic pathways, ion channels, myelination, mitochondrial energetics, cellular tight junction gene expression, as well as in multiple neuron and synapse-specific genes. Strikingly, in some circumstances rTMS had strong anti-inflammatory effects with broad down regulation of pathways involved in the classical complement cascade, Toll-like receptors, and other inflammatory pathways. This global transcriptional analysis induced by rTMS stimulation provides a baseline characterization for further experimental analysis and exploring potential clinical applications.

## Materials and methods

### Animals

Long–Evans rats (Charles River Laboratories), used for the *ex vivo* and *in vivo* experiments, were individually housed and maintained under specific pathogen-free conditions on a 12-h light/dark cycle at the National Institute on Aging/National Institute on Drug Abuse (NIA/NIDA) animal facilities in the Biomedical Research Center (Baltimore, MD). Standard rat chow and water were available *ad libitum* throughout the experiments. All procedures were approved by the Animal Care and Use Committee of the Intramural Research Program of the NIA.

### Background behavioral characterization

To establish the baseline cognitive status of the animals used in the *ex vivo* experiment, rats were tested in a ‘place’ version of the Morris water maze task, as previously described (Gallagher et al., 1993). The Morris water maze is a widely recognized tool for investigating spatial memory and learning in rodents, and the procedure used here has been extensively validated as a test for neurocognitive aging (Rapp et al., 1987; Rapp and Gallagher, 1996; Haberman et al., 2012; Gallagher et al., 2015; Tomás Pereira and Burwell, 2015).

Training continued over 8 consecutive days, three training trials per day. Every other day, the third trial was a probe in which the platform was inaccessible for 30 s. A learning index score was calculated for each animal from their average proximity to the escape platform during training; lower scores indicate better task performance. Aged rats that performed on par with young animals were denoted aged unimpaired (AU), while rats that scored greater than the young were classified as aged impaired (AI; Spiegel et al., 2013; Supplementary Figure 1). To control for non-mnemonic deficits, rats were tested in a single session of a hippocampus-independent cued water maze protocol the following day. No animals that performed outside the normal range on this version of the task were included in the present experiments.

### Tissue preparation

#### In vitro

Primary cultures of hippocampal neurons were prepared using hippocampi collected from E18 Sprague Dawley rats as previously described (Mazucanti et al., 2018). Dissociated cells were counted and plated (10^6^ cells/dish) in polyethyleneimine (Sigma-Aldrich) pre-coated coverslips on 35 mm dishes (zero days *in vitro*). Neurons were maintained in Neurobasal medium (GIBCO) supplemented with B27 (GIBCO), 2 mM glutamine, 100 U/ml penicillin, 100 μg/ml streptomycin, and 0.25 μg/ml amphotericin B. The cultures were placed in an incubator with extra insulation to prevent CO^2^ loss and decrease in temperature. The plates were placed centered directly on the inverted coil and received either 1 Hz, intermittent Theta Burst Stimulation (iTBS), or sham stimulation. Stimulation intensity was set at 15% of the machine’s maximum output (stimulation intensity for *in vitro* and *ex vivo* experiments was chosen as the average intensity for all groups in the *in vivo* experiment, detailed below). Neuronal cultures received either 1 Hz, iTBS, or sham stimulation. For the sham treatment, the plate was placed in the incubator with the coil approximately 20 cm away. Following stimulation, the plates were returned to the home incubator without the coil. RNA was isolated at 0, 2, 8 24, and 48 h after the end of stimulation.

#### Ex vivo

Four young (5–6 months), 4 AU, and 4 AI (24–25 months) male Long-Evans rats were sacrificed. Under RNase-free conditions, brains were removed, and the hippocampi were isolated and placed in cold, artificial cerebrospinal fluid (aCSF; 120 mM NaCl, 2.5 mM KCl, 1 mM NaH_2_PO_4_, 26 mM NaHCO_3_, 1.3 mM MgSO_4_, 10 mM D-Glucose, 3.3 mM CaCl_2_) saturated with 100% O_2_. The hippocampi were then quickly cut into approximately 7 to 9, 1 mm-thick slabs using a McIlwain tissue chopper (Pt#: MTC/2E; The Mickle Laboratory Engineering Co.), yielding approximately 15–18 hippocampal slices for each brain. Serial slices were distributed equally into 3 separate dishes (1 Hz, iTBS, sham) and incubated in fresh aCSF for 1 h at 32°C before administering rTMS. Hippocampal slices from young, AU, and AI rats were arranged 1 cm under the center of the coil and received either 1 Hz, iTBS, or sham stimulation, with the stimulation intensity set to 15% of the machine’s maximum output. Sham stimulation was applied to hippocampal slices arranged 20 cm from the coil. Hippocampal slices from one given animal were equally distributed across conditions (1 Hz, iTBS, and sham) and rested in aCSF for 2 h before RNA collection.

#### In vivo

Before rTMS administration, eight aged (26–27 months, 714–1,150 g) and eight young male rats (6–7 months, 502–788 g) were lightly anesthetized with isoflurane to reduce restraint stress and head movement during the stimulation procedure. The rats then received an intra-muscular injection of Dexmedetomidine (aged: 0.03 mg kg^−1^ body weight; young: 0.035 mg kg^−1^ body weight, the minimum required to prevent movement throughout the stimulation period). Antisedan (aged: 0.03 mg kg^−1^ body weight; young: 0.035 mg kg^−1^ body weight) was given after stimulation to reverse the sedation. Physiological parameters (heart rate, arterial blood oxygen saturation, body temperature) were monitored during anesthesia (Starr Life Sciences MouseOx Plus, Starr Life Sciences Corp. Oakmont, PA, USA) to ensure light and consistent anesthetic depth throughout stimulation. Rats were positioned on a heating pad and body temperature was maintained within 2°C of the initial measurement.

Young and aged rats received either iTBS or sham stimulation. Similar to procedures described in previous studies (Trippe et al., 2009; Mix et al., 2010, 2015; Benali et al., 2011; Hoppenrath et al., 2016), the coil was centered 8 mm above the rat’s skull, oriented with the handle to the left of the rat to produce a mediolaterally oriented electric field, aimed towards maximally stimulating the axons of the corpus callosum. The rat’s head was elevated with a small plastic conical ramp to ensure the skull was roughly parallel with the coil base and to minimize head movement and off-target body stimulation. Stimulator output intensity was adjusted to just below the level that elicited muscle twitching in the neck and head [aged: 15.0 ± 0.9% (11–17%); young: 15.3 ± 1.4% (12–19%) of maximal stimulator output], consistent with earlier preclinical studies in rats (Hoppenrath and Funke, 2013; Mix et al., 2015). Sham stimulation was performed with the coil 20 cm away from the rats’ heads.

Forty-eight hours post-stimulation animals in all conditions were deeply anesthetized with 5% isoflurane and sacrificed. Under RNase-free conditions, the brains were removed and freshly microdissected areas of the neocortex and dorsal hippocampus under the center of the TMS coil during stimulation were immediately frozen and stored at −80°C.

### rTMS protocols

rTMS was applied using a Magstim Rapid^2^ stimulator with a 70-mm figure-eight coil (The Magstim Company, Whitland, Dyfed, UK). 1 Hz stimulation was applied in 5 blocks of 600 pulses lasting 10 min repeated at 15-min intervals for a total of 3,000 pulses in 70 min. iTBS was administered in 5 blocks repeated every 15 min with each block consisting of 20 trains of 3 50-Hz pulse bursts repeated at 5 Hz for 2 s with a 10-s inter-train interval as described elsewhere (Huang et al., 2005). Each iTBS block consisted of 600 pulses in 192 s for a total of 3,000 pulses delivered in 63.2 min.

### Genome-wide gene expression analysis

#### RNA extraction

Total RNA was extracted by adding frozen individual hippocampi or hippocampal sections into prechilled tubes containing 1.0 mm glass beads (BioSpec Products, Bartlesville, OK) and RLT buffer and homogenized with a single 30 s 5,500 rpm cycle on a Precellys 24 homogenizer (Bertin Corp., Rockville, MD). The homogenate was centrifuged at 10,000 rpm for 10 min, the cleared lysate was transferred to a new tube, and RNA was column-purified according to the Qiagen RNeasy mini protocol (Qiagen, Germantown, MD). RNA concentration and quality were measured by Nanodrop (ThermoFisher, Waltham, MA USA) and the Agilent Bioanalyzer RNA 6000 Chip (Agilent, Santa Clara, CA).

#### Agilent microarray experiments

Two-hundred ng total RNA was labeled using the Agilent one-color Low-Input QuickAmp Labeling Kit (5190-2305, Agilent, Santa Clara, CA), purified on Qiagen columns, and quantified according to the manufacturer’s recommendations. A total of 600 ng Cy3-labeled cRNA was hybridized for 17 h to Agilent SurePrint G3 Rat Gene Expression v2 8x60K oligo microarrays (G4858-074036). Following post-hybridization rinses, arrays were scanned using an Agilent SureScan microarray scanner at 3-micron resolution, and hybridization intensity data was extracted from the scanned images using Agilent’s Feature Extraction Software. Raw and normalized microarray data have been deposited in the GEO data repository as SuperSeries GSE230150, with SubSeries GSE230147 (*in vitro*, *N* = 56 samples), GSE230148 (*ex vivo*, *N* = 40 samples), and GSE230149 (*in vivo*, *N* = 56 samples).

#### Microarray data analysis

The resulting dataset was analyzed with DIANE 6.0, a JMP microarray analysis program. The results were normalized with a z-score transformation (Cheadle et al., 2003). Z-normalized data were then analyzed with principal component analysis and sample hierarchical cluster to investigate the possible outliner samples and global genotype/treatment effects. To determine the gene expression changes within each specific RNA comparison, we first filtered probes by ANOVA test, then the pairwise statistical analysis is done by the z-test between different investigated groups with multiple comparison correction. The significant probes are determined by the cut off (Wagner et al., 2007) one way ANOVA *p* < 0.05 (Ferreri and Rossini, 2013) the z-test *p* < 0.05 and false discovery rate < 0.30 (Cavaleri et al., 2017) |z-ratio| > 1.5 (Cheng et al., 2017) average z-score for the pairwise sample > 0 (Cheadle et al., 2003). In other words, every differential expression effect of rTMS reported throughout this manuscript meets these statistical selection criteria.

#### Network analysis

The entire expression changes (z-ratio) result for each comparison are used as input. Gene set analysis using GO gene sets with the Parametric Analysis of Gene Set Enrichment (PAGE) algorithm was performed as previously described (Kim and Volsky, 2005). Protein interaction diagrams were generated from significant differentially expressed genes with the STRING interaction database. Functional grouping is denoted by colors, network nodes represent proteins and edges represent protein–protein relationships.

In addition to gene sets for functional gene groups, gene set analysis was also performed using highly specific Gene-Disease Associations (GAD) database (De et al., 2010; Zhang et al., 2010), in which every gene in each gene set has been statistically associated with a specific human disease or disorder through a population-based genetic association study. Noteworthy, an increase or decrease in a specific disease gene set simply means the aggregate values of the genes that have been associated with that disease have increased or decreased in expression, not that the disorder itself has increased or decreased or can be altered by rTMS treatment.

## Results

**Figure 1:**
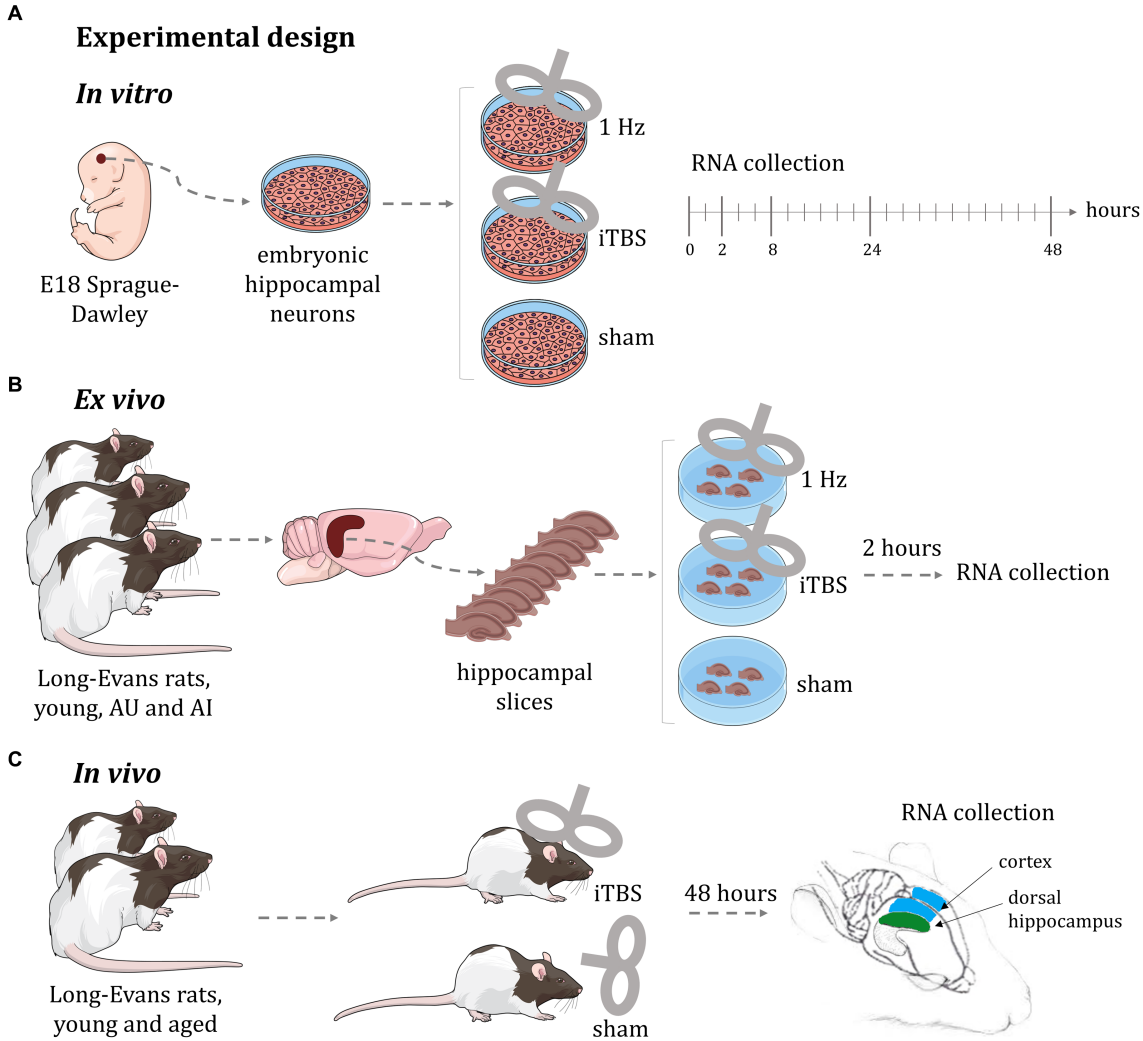
Experimental design of rTMS treatment. **(A)** *In vitro*: purified rat embryonal hippocampal neuronal cultures allowed identification of transcriptional changes over a 48-h time course using two stimulation protocols (1 Hz and iTBS); **(B)** *ex vivo*: hippocampal slices from a well-established rat model of aging enabled testing two different stimulation protocols (1 Hz and iTBS), in a short-term response model (2 h) across cognitive status in the context of complex cellular organization; **(C)** *in vivo*: young and aged animals allowed the identification of the global transcriptional response to rTMS, in a long-term response model (48 h), in two different brain regions (hippocampus and cerebral cortex), across age. AU: aged unimpaired; AI: aged impaired; iTBS: intermittent theta burst stimulation. This figure was in part created with BioRender software (BioRender.com).

In this global microarray-based gene expression analysis we used three distinct experimental modalities (Figure 1) to identify gross transcriptional changes induced by rTMS, each having specific advantages and limitations; (a) *in vitro*: purified rat embryonal hippocampal neuronal cultures allowed identification of transcriptional changes over a 48-h time course using two stimulation protocols (1 Hz and iTBS); (b) *ex vivo*: hippocampal slices from a well-established rat model of aging (Gallagher et al., 1993; Gallagher and Rapp, 1997) enabled testing two different stimulation protocols (1 Hz and iTBS), in a short-term response model (2 h) across cognitive status in the context of complex cellular organization; and (c) *in vivo*: young and aged animals allowed the identification of the global transcriptional response to rTMS, in a long-term response model (48 h), in two different brain regions (hippocampus and cerebral cortex), across age. In this way, we identified rTMS-induced transcriptional changes in multiple contexts. As a starting point for hypothesis generation, this report focuses on the effects of rTMS within each model and group, rather than on comparisons between models, cognitive status, or age groups. Detailed results of the transcriptional changes due to rTMS treatment in the context of age, cognition, and brain region will be presented elsewhere. Here the effects of TMS are evaluated as differential gene expression in contrasts between stimulated samples and corresponding sham controls within each experimental condition.

### rTMS produces broad transcriptional changes

**Figure 2:**
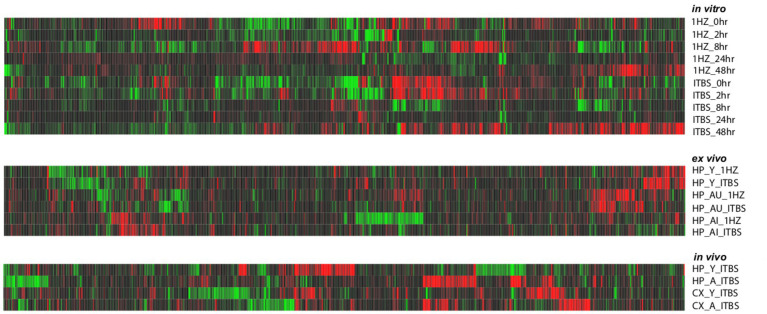
Heat map of gene expression changes due to rTMS treatment in three experimental models. Expression changes for individual genes were considered significant if they met four criteria: z-ratio > 1.5 or below −1.5; false detection rate < 0.30; a *p*-value statistic for z-score replicability <0.05; and mean background-corrected signal intensity > than zero. Red denotes higher relative expression and black to green are lower relative expression. This analysis suggests that rTMS treatment produces strikingly different patterns of gene expression across conditions within each model compared to their respective sham treatments.

As shown in Table 1, stimulation resulted in hundreds of significant transcriptional changes between rTMS and sham-treated controls in all experimental models, with both increases and decreases in each treatment group. While there are considerable overlaps between rTMS treatments, each sample-treatment combination produced a complex pattern of transcriptional response (Figure 2). This was evident in the *in vitro*, *ex vivo*, and *in vivo* experimental models. The transcriptional response was quite dynamic, often with both increases and decreases in the same gene and gene families with time, stimulation protocol, and relative to brain region. The complete set of statistically significant transcriptional changes due to rTMS can be found here (Supplementary Table 1).

**Table 1:** Number of genes changed in each rTMS group compared to its respective sham group.

Group	Upregulated	Downregulated
*In vitro*		
0 h 1 Hz	498	564
2 h 1 Hz	765	1,080
8 h 1 Hz	297	287
24 h 1 Hz	1,121	84
48 h 1 Hz	580	668
0 h iTBS	661	486
2 h iTBS	147	217
8 h iTBS	271	248
24 h iTBS	447	441
48 h iTBS	1,115	1,283
*Ex vivo*		
Young 1HZ	676	391
Young iTBS	593	436
AU 1HZ	532	433
AU iTBS	350	254
AI 1HZ	476	833
AI iTBS	687	432
*In vivo*		
Young cortex iTBS	78	253
Young hipp iTBS	229	165
Aged cortex iTBS	9	29
Aged hipp iTBS	34	21

iTBS, intermittent theta burst stimulation; AU, aged unimpaired; AI, aged impaired.

### rTMS alters the transcription of genes involved in complex neural processes

Table 2 (*in vitro*), Table 3 (*ex vivo*), and Table 4 (*in vivo*) show groups of selected genes with altered transcription (|z-ratio| > 1.5) implicated in complex neuronal processes in all three models tested. The categories of these genes include neurotransmission, synaptic function, inflammation, myelination, and cell–cell adhesion among other functional gene classes.

**Table 2:** *In vitro* model selected genes of rTMS induced alterations.

Gene symbol	1 Hz 0 h	1 Hz 2 h	1 Hz 8 h	1 Hz 24 h	1 Hz 48 h	iTBS 0 h	iTBS 2 h	iTBS 8 h	iTBS 24 h	ITBS 48 h	Gene name
GABA signaling											
*Gabbr1*	**−1.5**	**−2.8**	−0.4	**5.3**	0.9	0.1	−1.2	**−4.3**	0.5	−0.4	Gamma-aminobutyric acid type B receptor subunit 1
*Gabbr2*	1.1	**−1.7**	1.0	**1.5**	**1.8**	1.2	−0.5	1.0	**8.7**	−1.3	Gamma-aminobutyric acid type B receptor subunit 2
*Gabra4*	−1.0	−1.0	1.0	**1.7**	**2.5**	−0.8	**−4.1**	1.1	**2.3**	0.6	Gamma-aminobutyric acid type A receptor alpha4 subunit
*Gabrb1*	**−2.1**	−0.4	**1.6**	−0.6	**−2.5**	−0.9	1.0	0.9	**1.9**	**−7.9**	Gamma-aminobutyric acid type A receptor beta 1 subunit
*Gabrd*	**−5.0**	−1.3	1.0	**2.1**	**−11.4**	−1.0	**−2.3**	0.3	**34.6**	**−33.2**	Gamma-aminobutyric acid type A receptor delta subunit
*Gabrq*	0.3	−1.1	**1.7**	**−4.0**	**−4.5**	**2.6**	−0.2	**−2.9**	1.1	**11.9**	Gamma-aminobutyric acid type A receptor theta subunit
*Gabrg2*	−1.2	**−8.2**	1.0	0.9	0.2	**−2.1**	−1.0	0.4	0.8	**−15.7**	Gamma-aminobutyric acid type A receptor gamma 2 subunit
*Gabarapl1*	**1.9**	−0.2	**1.5**	**5.7**	**−5.9**	−1.0	0.0	**2.6**	−0.1	**−2.8**	GABA type A receptor associated protein like 1
*Gabarapl2*	**3.4**	0.4	0.4	0.6	**11.9**	−1.4	0.4	−0.2	−0.9	−0.2	GABA type A receptor associated protein like 2
*Atg4b*	0.2	−1.2	**1.6**	−1.0	**−3.0**	0.5	0.0	0.4	**5.3**	**−3.5**	Autophagy related 4B, cysteine peptidase
Glutamate signaling											
*Grik1*	**−4.0**	**−2.8**	1.3	**3.5**	−0.6	**−3.3**	0.0	0.1	**3.7**	**−2.5**	Glutamate ionotropic receptor kainate type subunit 1
*Grik4*	−1.3	**−4.0**	**2.9**	0.3	−1.0	−1.3	−0.3	0.6	**14.9**	−0.7	Glutamate ionotropic receptor kainate type subunit 4
*Gria3*	**2.7**	−0.8	−0.4	1.3	0.9	−0.8	**−2.3**	0.7	**3.0**	−1.1	Glutamate ionotropic receptor AMPA type subunit 3
*Grin1*	0.0	−0.3	0.3	**−2.0**	−1.3	**3.3**	0.5	0.0	0.7	**2.1**	Glutamate ionotropic receptor NMDA type subunit 1
*Grin2a*	−0.4	**3.8**	−0.6	1.1	1.3	−0.9	−0.4	**1.8**	0.9	**4.7**	Glutamate ionotropic receptor NMDA type subunit 2A
*Grin2c*	−1.4	**−1.9**	−0.5	**5.6**	**−2.0**	**−2.2**	−1.0	−0.2	−0.7	−0.1	Glutamate ionotropic receptor NMDA type subunit 2C
*Grin2d*	−1.3	**−1.5**	**7.9**	−0.8	**−4.5**	0.6	0.5	1.2	0.4	−1.3	Glutamate ionotropic receptor NMDA type subunit 2D
*Grm3*	−0.5	**−2.1**	**8.4**	**29.0**	1.4	**−1.8**	**−3.7**	**2.9**	0.8	**2.2**	Glutamate metabotropic receptor 3
*Grm5*	**−3.2**	−0.5	**1.8**	0.6	0.8	**−2.2**	0.2	1.1	1.3	**5.2**	Glutamate metabotropic receptor 5
*Grm6*	−0.4	**4.1**	0.4	−1.3	**2.9**	**4.0**	0.7	−1.2	−1.1	**5.8**	Glutamate metabotropic receptor 6
*Grm7*	0.6	**−11.0**	−0.3	**3.5**	−0.1	**−1.8**	**−1.6**	0.1	0.4	**−3.2**	Glutamate metabotropic receptor 7
*Cacng3*	**−1.6**	**−1.5**	**4.4**	**−1.9**	**−3.5**	0.1	−0.1	−1.3	1.1	1.4	Calcium voltage-gated channel auxiliary subunit gamma 3
*Calm1*	**5.0**	−0.8	0.7	1.1	**2.1**	**−2.6**	−0.4	**2.2**	**2.3**	**3.0**	Calmodulin 1
*Camk2b*	−0.3	**−4.6**	**1.9**	**7.3**	**−2.1**	−0.2	**−2.2**	**1.6**	**2.5**	−0.5	Calcium/calmodulin-dependent protein kinase II beta
*Camk2g*	**4.0**	**−6.7**	−0.6	**82.1**	−0.7	**−1.9**	**−1.5**	1.1	−0.3	**−3.6**	Calcium/calmodulin-dependent protein kinase II gamma
*Ppp3ca*	−0.2	−0.8	1.0	0.1	1.2	−0.3	−0.7	0.4	**1.8**	**4.1**	Protein phosphatase 3 catalytic subunit alpha
Kegg: learning and memory											
*Casp3*	−1.0	**−9.4**	**2.4**	**−3.9**	**−4.5**	**−5.1**	−0.1	**2.0**	0.4	0.7	Caspase 3
*Comt*	**−4.4**	**−4.3**	**1.7**	−0.5	0.6	0.0	−0.5	**7.2**	**2.0**	**2.4**	Catechol-O-methyltransferase
*Creb1*	1.1	0.9	**−6.4**	**1.9**	**1.8**	1.4	−0.4	−1.0	−0.7	**−1.8**	cAMP Responsive element binding protein 1
*Fgf13*	**3.2**	−0.8	**5.1**	0.7	−0.5	**−1.6**	0.1	**1.8**	−0.9	**−1.6**	Fibroblast growth factor 13
*Gpi*	−0.8	**−2.4**	**4.7**	−0.4	**−4.8**	−1.1	0.6	1.5	0.7	**1.5**	Glucose-6-phosphate isomerase
*Igf1*	0.5	**2.7**	**1.8**	**−7.8**	1.4	**5.4**	0.8	**−3.7**	1.3	**1.6**	Insulin-like growth factor 1
*Ngf*	1.1	**3.5**	0.5	**−1.5**	**−4.7**	**5.0**	0.3	0.1	0.5	**6.0**	Nerve growth factor
*Prkca*	0.1	1.2	**−2.0**	**1.7**	0.0	−0.4	0.1	0.5	**27.2**	**−4.9**	Protein kinase C, alpha
*Prkcz*	0.1	**−3.2**	0.8	−0.5	**1.5**	0.3	1.0	−1.1	**−1.5**	1.0	Protein kinase C, zeta
*Ptprz1*	−0.4	**−1.5**	**−2.9**	−0.5	0.3	**−1.5**	−1.3	−1.3	0.8	**10.9**	Protein tyrosine phosphatase, receptor type Z1
*Reln*	**6.3**	**−8.3**	**−2.1**	**2.1**	−0.8	0.0	−0.1	1.2	−0.1	**−1.5**	reelin
*Shank3*	1.1	**−3.0**	−0.4	−0.5	**−6.9**	1.0	**1.7**	1.2	**−3.9**	**6.0**	SH3 and multiple ankyrin repeat domains 3
*Th*	**1.5**	**1.7**	−0.3	**−9.4**	−1.1	**3.7**	0.3	**−3.2**	0.2	**2.0**	Tyrosine hydroxylase
*Trpm7*	0.3	**6.1**	0.5	**−3.6**	0.9	**6.0**	1.0	**−5.1**	0.4	**3.0**	Transient receptor potential cation channel, subfam M, 7
Kegg: Alzheimer’s disease											
*Apbb1*	**−2.2**	**−17.3**	**4.0**	**2.8**	1.3	1.2	0.4	−0.2	−0.7	**4.4**	Amyloid beta precursor protein binding family B member 1
*Apoe*	**−1.8**	**−2.0**	1.2	**2.3**	**−34.5**	**−1.8**	**−1.7**	**1.9**	0.2	−1.2	Apolipoprotein E
*Atf6*	0.6	**7.5**	**−3.0**	**1.9**	0.9	0.0	−1.4	1.3	**4.4**	**−4.5**	Activating transcription factor 6
*Atp2a2*	0.0	0.3	**−2.2**	1.2	**−12.8**	1.3	−0.1	**−1.8**	**5.7**	**−1.5**	ATPase sarcoplasmic/endoplas. Retic. Ca^2+^ transporting 2
*Bad*	−0.3	−0.7	1.3	−0.3	−0.2	**2.3**	−0.2	0.4	−1.2	**−2.0**	BCL2-Associated agonist of cell death
*Casp3*	−1.0	**−9.4**	**2.4**	**−3.9**	**−4.5**	**−5.1**	−0.1	**2.0**	0.4	0.7	caspase 3
*Eif2ak3*	−0.2	**3.9**	−0.8	0.5	−0.9	−0.2	**−2.6**	1.0	1.2	**−9.5**	Eukaryotic translation initiation factor 2 alpha kinase 3
*Gsk3b*	−0.9	−0.5	**4.0**	−1.1	−0.9	−0.2	1.4	0.9	−0.1	0.9	Glycogen synthase kinase 3 beta
*Mapt*	**2.8**	**−3.8**	1.3	**2.4**	0.7	**6.6**	−0.5	**3.9**	**−5.9**	**−3.6**	Microtubule-associated protein tau
*Ppp3cb*	−0.5	−0.5	0.4	**4.9**	0.7	−0.5	**−7.7**	1.2	0.4	**2.8**	Protein phosphatase 3 catalytic subunit beta
*Psen2*	**−1.6**	**−3.5**	**−1.5**	0.0	**−12.5**	0.1	−1.0	−0.1	**2.5**	−1.2	Presenilin 2
*Tnfrsf1a*	0.3	0.3	**−2.7**	−0.4	0.8	**11.4**	**−4.1**	−0.1	**3.5**	**−2.1**	TNF receptor superfamily member 1A

In bold, genes with z-ratio > 1.5, *p* value < 0.3 (false discovery rate correction), p value statistic for z-score replicability below 0.05, and mean background-corrected signal intensity > 0. Bold values represent z-ratio.

**Table 3:** *Ex vivo* model selected genes of rTMS induced alterations.

Gene symbol	Y 1HZ	Y iTBS	AU 1HZ	AU iTBS	AI 1HZ	AI iTBS	Gene name
Complement							
*C1qc*	**−2.29**	**−3.15**	0.13	1.02	0.14	**1.59**	Complement C1q C chain
*C1qb*	**−2.25**	**−1.96**	−0.15	−0.60	−0.42	**2.06**	Complement C1q B chain
*C2*	**−2.58**	**−3.36**	−0.18	0.73	0.68	**2.92**	Complement C2
*C3*	−1.19	**−3.75**	−0.33	1.12	0.19	**3.01**	Complement C3
*C3ar1*	**−1.90**	**−2.26**	0.49	0.81	−0.33	−0.34	Complement C3a receptor 1
*C4a*	**−3.96**	**−3.62**	−0.37	1.05	−0.03	**2.68**	Complement C4A
*C4b*	**−3.69**	**−3.57**	−0.02	0.56	−0.14	**2.94**	Complement C4B
*Cfhr1*	**−1.97**	−1.34	−0.48	−1.13	**2.17**	−1.27	Complement factor H-related 1
Inflammatory							
*Il12b*	**−3.83**	**−3.60**	−0.17	0.55	1.19	**2.72**	Interleukin 12B
*Il1b*	**−3.71**	**−5.54**	−0.24	**−1.53**	−0.25	1.42	Interleukin 1 beta
*Il1rn*	**−3.22**	**−4.00**	−0.03	0.42	−0.24	0.80	Interleukin 1 receptor antagonist
*Il1a*	**−2.17**	**−3.76**	0.16	−0.77	−0.43	0.71	Interleukin 1 alpha
*Cd74*	**−3.79**	**−5.01**	−0.45	**1.98**	1.03	**4.65**	CD74 Molecule, MHC class II invariant chain
*Cd68*	**−3.31**	**−3.52**	−0.19	0.05	0.10	**2.51**	Cd68 Molecule
*Cxcl13*	**−2.89**	**−4.01**	−1.25	**−1.51**	**−1.72**	**3.46**	C-X-C Motif chemokine ligand 13
*Tnfsf9*	**−2.84**	**−3.13**	0.28	−0.78	**1.57**	**2.07**	TNF Superfamily member 9
Myelination							
*Mobp*	**2.05**	−0.05	0.09	**1.15**	−0.01	−0.43	Myelin-associated oligodendrocyte basic protein
*Mog*	**1.77**	−0.65	−0.15	1.06	0.80	−0.29	Myelin oligodendrocyte glycoprotein
*Cnp*	**1.61**	−0.49	**1.81**	**3.00**	0.55	−0.76	2′,3′-Cyclic nucleotide 3′ phosphodiesterase
*Sox10*	0.91	**−3.23**	0.39	1.14	0.34	−0.21	SRY box 10
*Erbb3*	1.42	0.02	0.34	**1.62**	0.90	−0.42	Erb-b2 receptor tyrosine kinase 3
*Tf*	**1.53**	0.32	0.67	1.14	0.26	0.18	Transferrin
*Cadm4*	0.49	**2.29**	−0.61	−0.67	0.25	−0.15	Cell adhesion molecule 4
*Klk6*	**1.90**	−0.04	0.52	1.34	0.95	−0.43	Kallikrein related-peptidase 6
Claudins							
*Cldn2*	**1.55**	1.16	0.14	1.19	−0.03	−0.99	Claudin 2
*Cldn4*	**3.21**	**2.38**	−0.18	0.61	**2.00**	**−1.93**	Claudin 4
*Cldn6*	**3.62**	0.65	−1.38	**−2.19**	**2.85**	**1.77**	Claudin 6
*Cldn7*	0.33	0.34	0.79	0.37	**2.03**	**2.53**	Claudin 7
*Cldn11*	**2.17**	0.24	0.51	1.28	0.47	0.08	Claudin 11
*Cldn14*	−0.65	−0.80	1.13	**1.50**	0.20	1.24	Claudin 14
*Cldn16*	−0.22	**3.13**	−1.29	**−3.42**	−0.87	0.16	Claudin 16
*Cldn20*	**1.73**	−0.15	0.91	1.01	**−1.69**	−1.43	Claudin 20
*Cldn23*	**−1.53**	**1.70**	0.18	−0.03	0.42	**−2.95**	Claudin 23
Neuronal genes							
*Gabra6*	−0.97	**1.96**	0.90	0.37	0.16	**1.97**	Gamma-aminobutyric acid (GABA) A receptor, alpha 6
*Sv2b*	−0.12	**1.71**	**2.01**	0.16	**1.92**	0.48	Synaptic vesicle glycoprotein 2b
*Grin3a*	**−1.85**	**2.19**	**2.33**	−0.46	−0.62	−0.48	Glutamate receptor, ionotropic, N-methyl-D-aspartate 3A
*Chrna5*	**−1.52**	**1.83**	−0.75	−0.84	0.43	−0.59	Cholinergic receptor, nicotinic, alpha 5 (neuronal)
*Slc6a13*	0.29	**1.79**	−0.61	−0.38	−0.08	−0.83	Solute carrier family 6 (neurotransmitter transporter), member 13
*Slc5a7*	**−2.67**	**−5.00**	−0.98	0.34	**3.00**	**3.25**	Solute carrier family 5 (sodium/choline cotransporter), member 7
Other							
*Plac8*	**−3.97**	**−3.17**	−1.18	−1.26	0.21	**4.46**	Placenta-specific 8
*Igf2*	1.22	**2.99**	−0.56	**−3.73**	−0.08	−1.06	Insulin-like growth factor 2
*Folr1*	**3.58**	**2.71**	**−4.41**	0.11	**1.88**	**−1.55**	Folate receptor 1 (adult)
*Otx2*	**6.83**	**2.35**	−1.40	**4.57**	**−1.57**	**−6.04**	Orthodenticle homeobox 2
*Slc22a7*	−0.75	−1.40	**−2.87**	0.75	**4.11**	**1.85**	Solute carrier family 22 (organic anion transporter), member 7

In bold, genes with z-ratio > 1.5, *p* value < 0.3 (false discovery rate correction), *p* value statistic for z-score replicability below 0.05, and mean background-corrected signal intensity > 0. Bold values represent z-ratio. Y, Young rats; AU, Aged-unimpaired rats; AI, Aged-impaired rats (for classification criterion, see Background behavioral characterization in Material and Methods).

**Table 4:** *In vivo* model selected genes of rTMS induced alterations.

Gene symbol	Y CX iTBS	A CX iTBS	Y HP iTBS	A HP iTBS	Gene name
Neuronal genes					
*Gas7*	**2.13**	1.09	**1.67**	−0.38	Growth arrest specific 7
*Grin2d*	**1.79**	**1.42**	**4.54**	0.45	Glutamate ionotropic receptor NMDA type subunit 2D
*Ryr2*	**2.80**	**1.58**	−0.51	−0.33	Ryanodine receptor 2
*Otof*	**2.55**	**1.68**	−0.58	−0.41	Otoferlin
*Sorl1*	**1.63**	0.41	−0.48	0.24	Sortilin related receptor 1
*Penk*	−0.88	**3.00**	−0.52	0.33	Proenkephalin
*Prodh1*	0.59	**2.51**	−0.62	−0.35	Proline dehydrogenase 1
*Ptk2b*	0.65	**2.67**	−0.97	1.40	Protein tyrosine kinase 2 beta
*Arc*	0.11	**−3.62**	0.32	**−1.89**	Activity-regulated cytoskeleton-associated protein
*S100b*	**−2.92**	**−3.15**	**−1.78**	**−2.95**	S100 calcium binding protein B
*Htr2c*	0.23	**−2.84**	1.15	**−1.68**	5-Hydroxytryptamine receptor 2C
*Grm2*	**1.66**	1.22	0.62	−0.29	Glutamate metabotropic receptor 2
*Rgs4*	0.61	0.53	1.39	**−3.12**	Regulator of G-protein signaling 4
*Nxph3*	0.68	−0.09	0.67	**−3.65**	Neurexophilin 3
Complement					
*C1s*	−0.05	0.19	**−2.02**	0.87	complement C1s
*C2*	−0.94	−0.21	**−1.60**	1.11	complement C2
Immune					
*RT1-Da*	**−3.60**	−0.53	**−3.55**	**3.19**	RT1 class II, locus Da
*Cd74*	**−3.23**	0.57	**−3.46**	**2.62**	CD74 molecule, MHC class II invariant chain
*Tlr3*	**−3.02**	**−2.29**	**−2.77**	−0.96	Toll-like receptor 3
*Irf7*	−0.09	**2.43**	−0.42	**2.34**	Interferon regulatory factor 7
Hemoglobin					
*Hbb*	−0.98	**−1.99**	1.33	**−2.40**	Hemoglobin subunit beta
*Hba1*	−0.45	**−1.64**	1.15	**−3.00**	Hemoglobin, alpha 1
*Hbe2*	−0.13	**−1.81**	0.93	**−3.03**	Hemoglobin, epsilon 2
*Hbb-b1*	0.56	1.26	**3.55**	**−2.83**	Hemoglobin, beta adult major chain
Other					
*Ttr*	**4.47**	**2.61**	**−2.86**	0.56	Transthyretin
*Bmp4*	−0.40	**2.17**	−1.01	**1.58**	Bone morphogenetic protein 4
*Klhl14*	0.35	**−4.93**	**1.50**	−0.84	Kelch-like family member 14
*Hook3*	−0.63	**−3.45**	0.65	**−2.76**	Hook microtubule-tethering protein 3
*Rxrg*	−0.19	**−3.06**	**2.23**	−0.70	Retinoid X receptor gamma
*Slc27a2*	**−4.07**	**−2.92**	−0.64	**−4.69**	Solute carrier family 27 member 2
*Abcg2*	**−4.47**	−0.36	**−2.50**	0.32	ATP-binding cassette, subfamily G (WHITE), member 2
*Prg2*	**5.78**	−0.28	**2.84**	−1.16	Proteoglycan 2
*Adam7*	**5.43**	**1.52**	**−1.73**	−0.67	ADAM metallopeptidase domain 7
*Prb1*	**4.97**	1.32	**3.46**	0.44	Proline-rich protein BstNI subfamily 1
*Mrpl43*	**4.48**	−0.15	**2.63**	0.08	Mitochondrial ribosomal protein L43
*Sema6c*	**1.62**	**1.64**	**4.28**	0.39	Semaphorin 6C
*S100b*	−1.30	**−2.99**	**−4.01**	**−2.64**	S100 Calcium binding protein B
*Ppp1r16b*	**−2.93**	**1.95**	**−2.14**	**2.17**	Protein phosphatase 1, regulatory subunit 16B
*Mis18a*	**−3.58**	**2.72**	**−3.16**	**4.22**	MIS18 kinetochore protein A
*Ranbp3l*	**−2.80**	**1.03**	**0.46**	**3.01**	RAN binding protein 3-like
*Enpp6*	**1.65**	**1.82**	0.06	**2.83**	Ectonucleotide pyrophosphatase/phosphodiesterase 6
*Gpat2*	0.35	1.21	**2.42**	**−3.62**	Glycerol-3-phosphate acyltransferase 2, mitochondrial
*Alas2*	0.03	**−2.07**	−0.08	**−3.75**	5′-Aminolevulinate synthase 2
*Apold1*	−0.77	**−3.41**	0.51	**−2.58**	Apolipoprotein L domain containing 1

In bold, genes with z-ratio > 1.5, *p* value < 0.3 (false discovery rate correction), *p* value statistic for z-score replicability below 0.05, and mean background-corrected signal intensity > 0. Bold values represent z-ratio. Y, Young rats; A, Aged rats.

**Figure 3:**
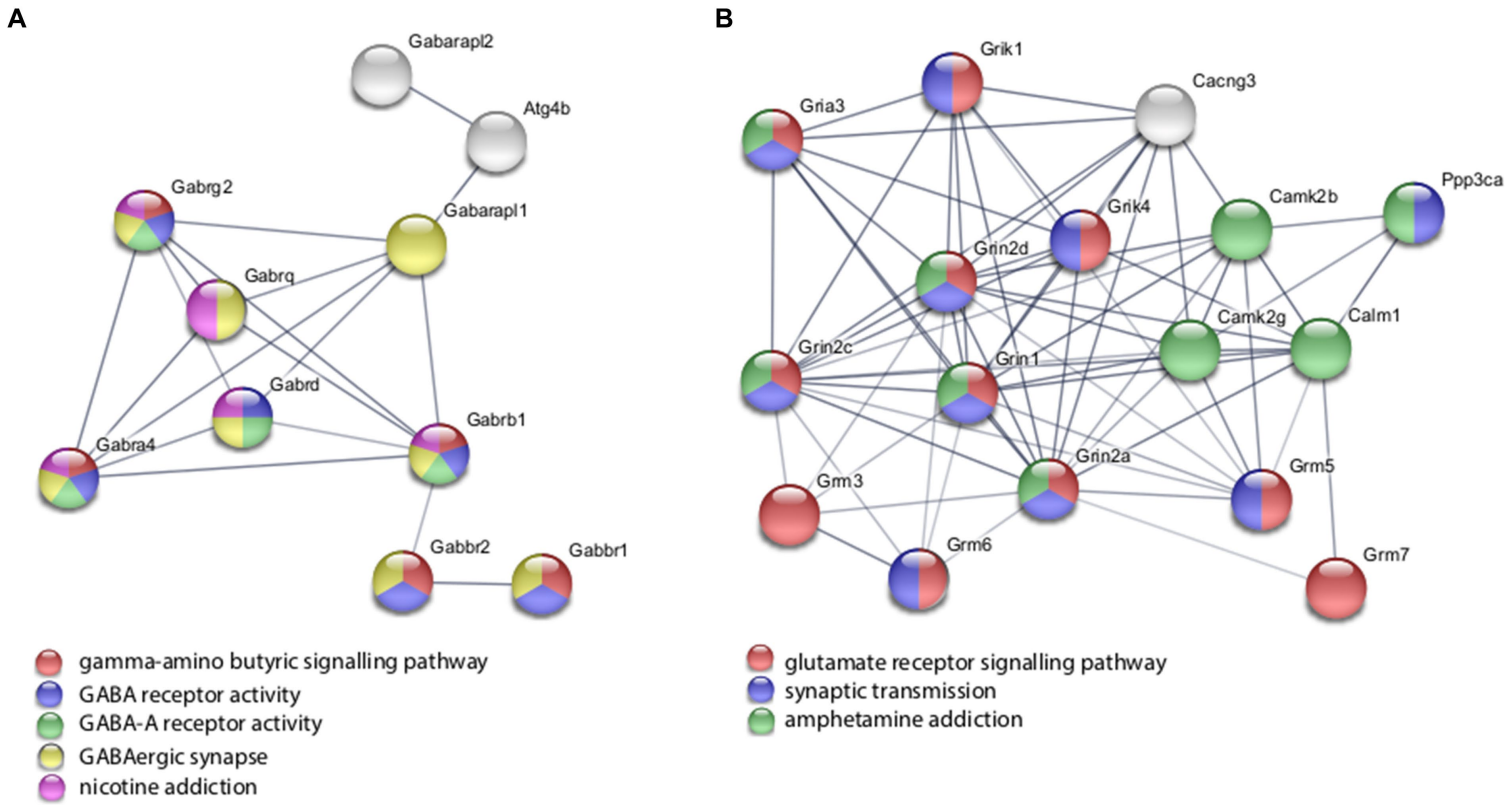
Selected protein–protein interaction network analysis from rTMS altered differential gene expression in the *in vitro* model. Networks built from shared genes involved in inhibitory and excitatory neuronal functions, including multiple **(A)** GABA and **(B)** glutamate receptors. Expression changes for individual genes were considered significant if they met four criteria: z-ratio > 1.5 or below −1.5; false detection rate < 0.30; a *p*-value statistic for z-score replicability <0.05; and mean background-corrected signal intensity > than zero. Functional or biological grouping is denoted by colors. Network nodes represent proteins and edges represent protein–protein relationships.

**Figure 4:**
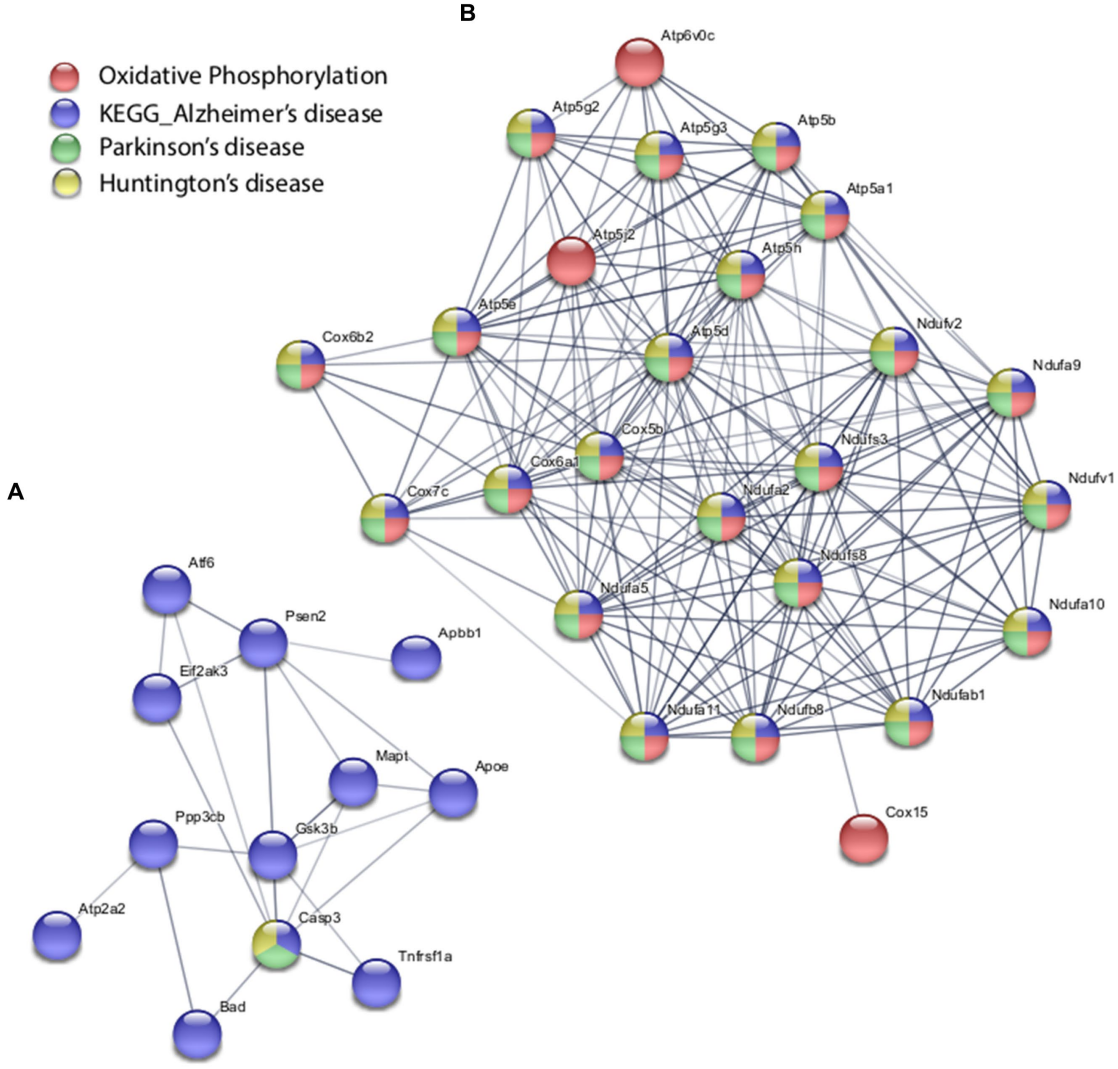
Selected protein–protein interaction network analysis from rTMS altered differential gene expression in the *in vitro* model. Networks built from shared genes involved in glutamate signaling and neurotransmission. **(A)** Transcriptional alteration of genes involved in Alzheimer’s disease. **(B)** Transcriptional response was altered in oxidative phosphorylation pathways including shared genes involved in Huntington’s, Alzheimer’s, and Parkinson’s disease. Expression changes for individual genes were considered significant if they met four criteria: z-ratio > 1.5 or below −1.5; false detection rate < 0.30; a *p*-value statistic for z-score replicability <0.05; and mean background-corrected signal intensity > than zero. Functional grouping is denoted by colors. Network nodes represent proteins and edges represent protein–protein relationships.

With the *in vitro* model using rat hippocampal neuronal cultures, genes involved in inhibitory and excitatory neurotransmission were altered, both upregulated and downregulated (Table 2), which included multiple GABA (Figure 3A) and glutamate receptors (Figure 3B) such as *Gabbr1,2*; *Grik1,4*; *Grm3-7*, and *Gabra4* (Heidelberg et al., 2013) as well as genes involved in learning and memory-related plasticity. In addition, the transcriptional response was altered in oxidative phosphorylation pathways including shared genes involved in Huntington’s, Alzheimer’s, and Parkinson’s disease (Figures 4A,B). In the *ex vivo* and *in vivo* models (Tables 3, 4) altered transcription of additional neuronal genes was shown to include genes involved in neurotransmission [*Ptk2b* (Brys et al., 2013), *Slc6a13* (Christiansen et al., 2007)]; choline transport [*Slc5a7* (Ribeiro et al., 2006), *Ryr2* (Abu-Omar et al., 2017), *Chrna5* (Proulx et al., 2014)]; synaptic function [*Grin3a* (*Glun3a*; Perez-Otano et al., 2016)]; plasticity (*Arc*; Tomas Pereira et al., 2015, *Cnp*; Barmashenko et al., 2014); learning and memory [*Arc*; Morin et al., 2015; Tomas Pereira et al., 2015), *Grin2d*, (*Glun2d*; Jacobs et al., 2014)], and cognition (*Ryr2*; Liu et al., 2012, *Arc*; Morin et al., 2015, *Sorl1*; Li et al., 2017), among others.

**Figure 5:**
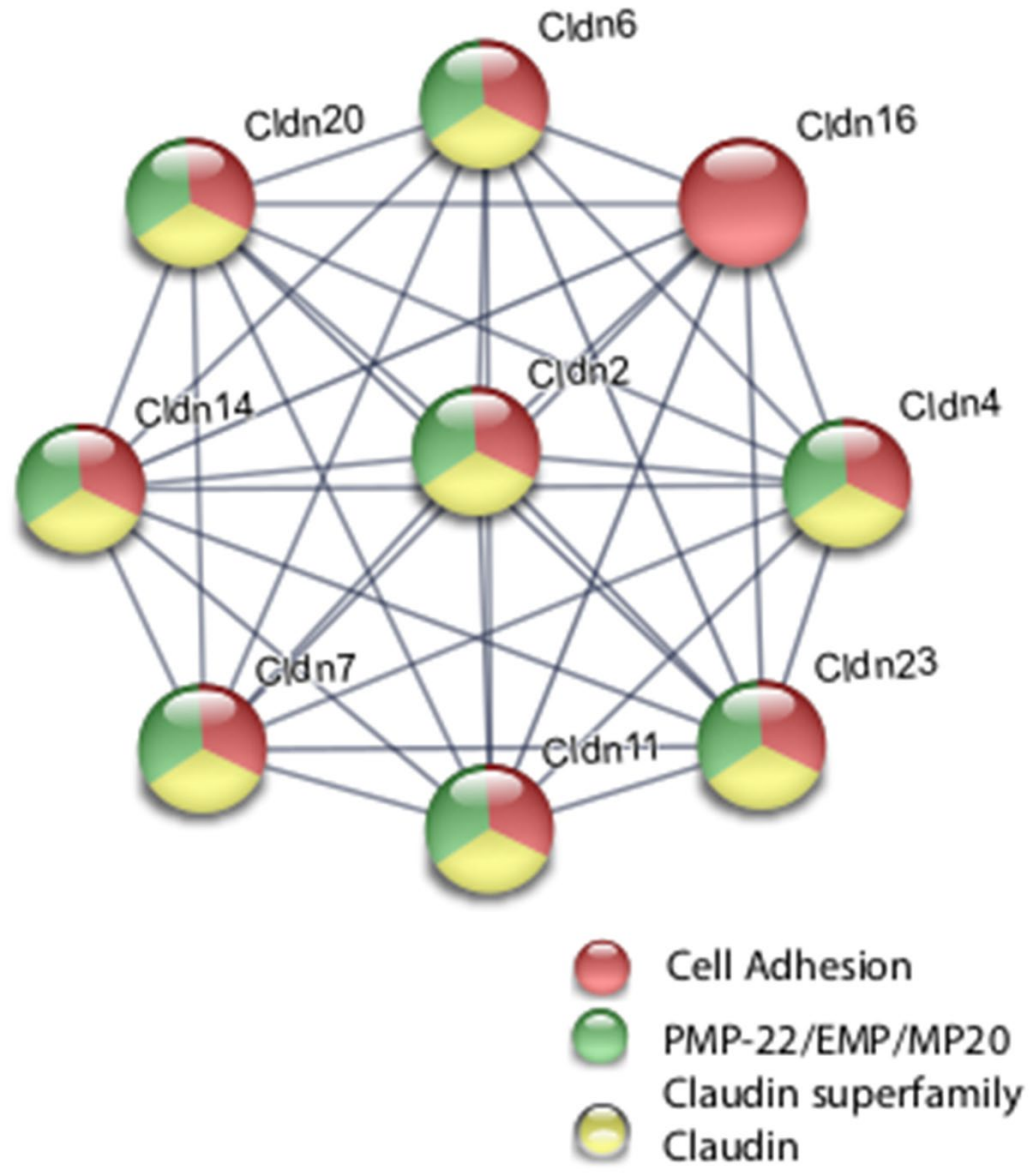
Selected protein–protein interaction network analysis from rTMS altered differential gene expression in the *ex vivo* model. Network built from shared genes involved in Claudin family members. Expression changes for individual genes were considered significant if they met four criteria: z-ratio > 1.5 or below −1.5; false detection rate < 0.30; a *p*-value statistic for z-score replicability <0.05; and mean background-corrected signal intensity > than zero. Functional grouping is denoted by colors. Network nodes represent proteins and edges represent protein–protein relationships.

Coordinate transcriptional upregulation occurred in a cohort of myelin regulatory genes at 2 h post-rTMS stimulation in the *ex vivo* model (Table 3). These include increases in *Mobp*, *Mog*, *Cnp*, *Erbb3 Klk6*, *Cadm4*, and *transferrin* (*Tf*). However, the myelin regulatory factor *Sox10* was downregulated in the iTBS-stimulated *ex vivo* samples. Myelin-related transcriptional induction was not statistically significant at 48 h for *in vivo* or at 2 h for *in vitro* cultures. Moreover, gene expression from nine Claudin family members was both increased and decreased in the *ex vivo* model (Table 3). Claudins mediate cell–cell contact and blood–brain barrier integrity. A shared protein domain network showing interrelationships between the TMS transcriptionally altered Claudins is shown in Figure 5.

### rTMS results in a broad-based anti-inflammatory transcriptional response

rTMS produced a substantial reduction in transcripts involved in immune and inflammatory processes in the *ex vivo* and *in vivo* samples. Most striking was the coordinate downregulation of many members of the classical complement pathway in the *ex vivo* rTMS model with both 1 Hz and iTBS (Table 3) stimulation after 2 h. In addition, the expression of complement factors *C1s* and *C2* was reduced in the *in vivo* model in the young hippocampus at 48 h (Table 4). In contrast, *C1qb*, *C1qc*, *C2*, *C3*, *C4a*, and *C4b* were *increased* in the AI hippocampus using iTBS in the *ex vivo* model (Table 3). Although generally thought of as mediators of innate immunity, members of the complement cascade have also been shown to be important in synaptic remodeling (Presumey et al., 2017), and have been associated with schizophrenia (Nimgaonkar et al., 2017), age-related macular degeneration (Lu et al., 2018), and Alzheimer’s disease (Torvell et al., 2021).

Additional inflammatory genes that were transcriptionally repressed by rTMS included *Il1b*, *Il1rn*, *Cd74*, and *Il12b* in the *ex vivo* model following iTBS (Table 3), and *Tlr3*, *Cd74*, and *RT1-Da* in the *in vivo* model (Table 4). Moreover, the gene for *S100b*, a marker of neuroinflammation and neuronal damage, was markedly downregulated by rTMS in all *in vivo* samples (Table 4). Importantly, immune regulatory molecules as a group were not significantly altered by rTMS in the *in vitro* model using purified neuronal cultures suggesting that modulation of immune transcripts in both *ex vivo* and *in vivo* models (both complex tissue intact samples) were from non-neuronal cells including resident glial cell populations.

### rTMS affects the transcription of genes implicated in disorders for which it is used clinically

Notably, expression was altered among multiple genes that have been studied in disorders for which rTMS is used clinically, including depression [*Slc6a4* (Lam et al., 2018), *S100b* (Schroeter et al., 2014), *Il18* (Prossin et al., 2011; Bufalino et al., 2013; Kim et al., 2017), *Il1b* (Bufalino et al., 2013), *Htr2c* (Brummett et al., 2014), *Gabra6* (Inoue et al., 2015)], epilepsy [*Grin2d* (*GluN2D*; Li D. et al., *2016*), *Gabra6* (Prasad et al., 2014), *Scn1b* (Ramadan et al., 2017), *Scn3a* (Lamar et al., 2017), *Kcnt1* (Evely et al., 2017)], schizophrenia [*Rgs4* (Schwarz, 2017), *Grin1* (Zhao et al., 2006), *Grm5* (Matosin et al., 2017), *Pde10a* (Boden et al., 2017), *C4a* (Nimgaonkar et al., 2017)], bipolar disorder [*Pde10a* (McDonald et al., 2012); *S100b* (da Rosa et al., 2016)], Parkinson’s disease [*Nr4a2* (Liu et al., 2017)], Alzheimer’s disease and other dementias [Ptk2b (Li Y. Q. et al., 2016), *Ttr* (Silva et al., 2017), *Nr4a2* (Montarolo et al., 2016), *Ryr2* (Briggs et al., 2017), *Mobp* (Irwin et al., 2014), *Cd40* (Giunta et al., 2010), *Grn* (Sudre et al., 2017), *Hmo1* (Sung et al., 2016), *Arc* (Bi et al., 2017)], stroke [*Lgals3* (*Gal3*; He et al., 2017), *Ace* (Wei et al., 2017), *Cd40* (Huang et al., 2017), *Cxcl12* (Shen et al., 2017), *Hmgb1* (Choi et al., 2017)], as well as substance abuse [*Slc6a4* (Bauer et al., 2015), *Penk* (Moeller et al., 2015), *Rgs4* (Ho et al., 2010), *Chrna5* (Lassi et al., 2016; Olfson et al., 2016)]. Overlap between the complex functional processes, disorders, and genes mentioned here highlights a central role of shared fundamental neuronal pathways in multiple processes and distinct neurological disorders (Brainstorm et al., 2018; Gandal et al., 2018).

Moreover, TMS altered the expression of genes studied in other disorders, suggesting additional potential clinical applications of TMS. These include hereditary motor neuropathy [*Slc5a7* (Barwick et al., 2012)], congenital myasthenic syndrome (*Slc5a7*; Bauche et al., 2016), age-related hearing loss (*Gabra6*; Sun et al., 2014), Canavan disease (*Gabra6*; Surendran et al., 2003), gout (*Abcg2*; Yu et al., 2017), pancreatitis (*Cldn2*; Giri et al., 2016), as well as numerous genes involved in inflammatory and immune disorders. Additionally, TMS alters the transcription of many genes of unknown function. These can be found in Supplementary Table 1.

### Gene set analysis of rTMS transcriptional changes

**Figure 6:**
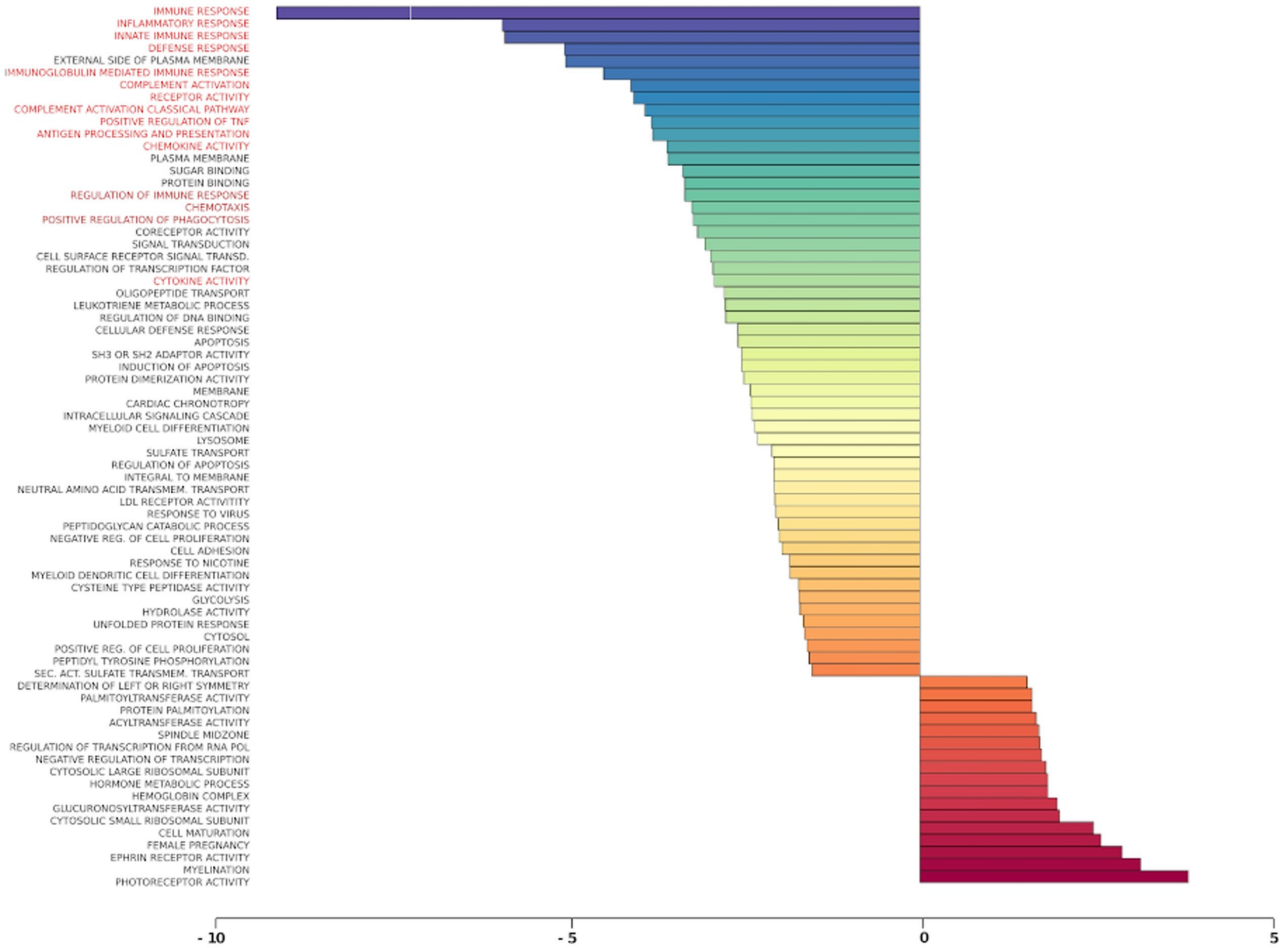
Gene ontology gene set analysis in the *ex vivo* model, 1 Hz stimulation protocol, young. Red text highlights gene groups involved in inflammation and immune response following 1 Hz stimulation of hippocampal slices from young rats compared to sham treatment. Significantly changed gene sets, associated with biological processes, are organized by z-score.

**Figure 7:**
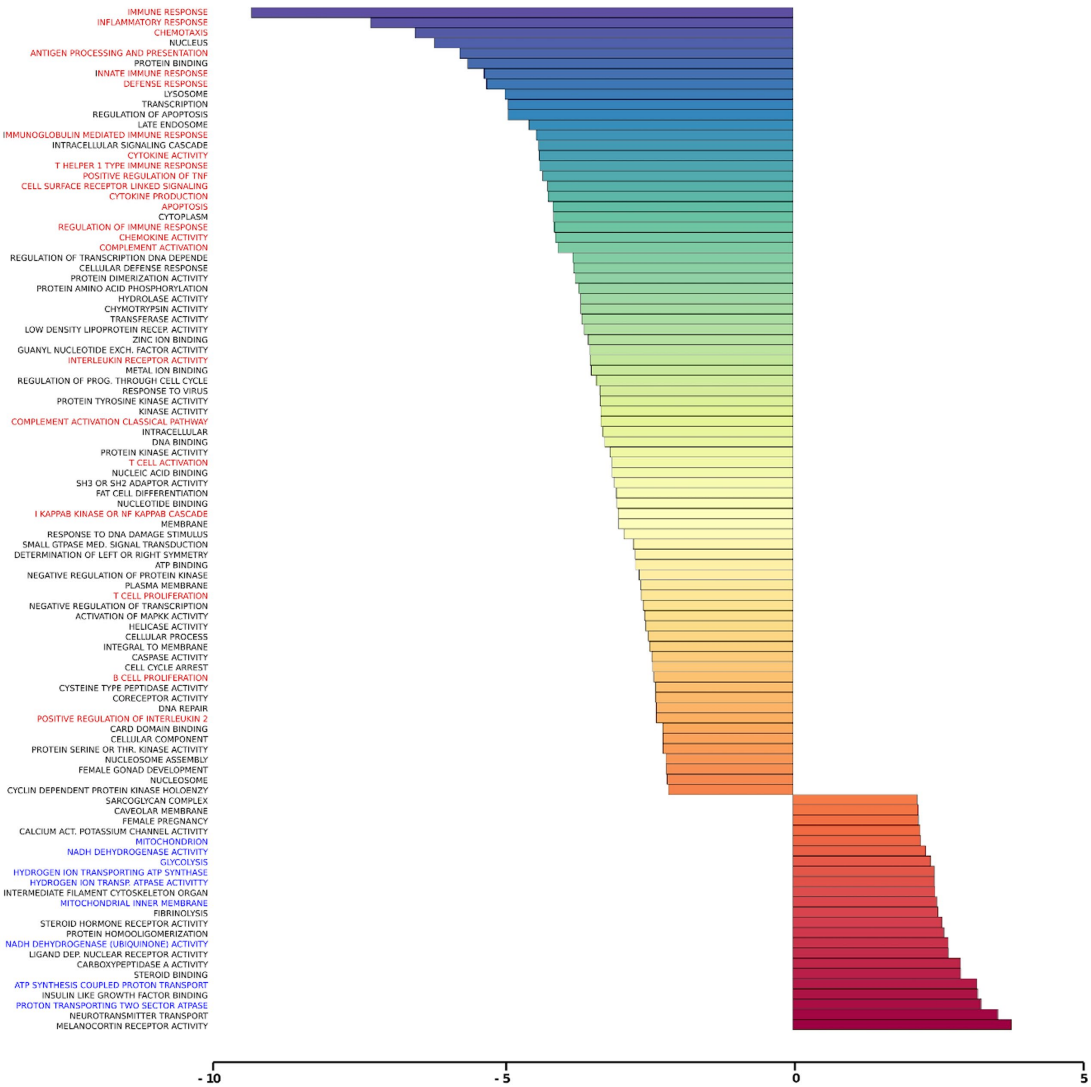
Gene ontology gene set analysis in the *ex vivo* model, iTBS protocol, young. Text highlighted in red indicates gene groups involved in inflammation and immune response, and blue text signifies gene groups involved in mitochondrial/energy processes following iTBS in hippocampal slices from young rats compared to sham treatment. Significantly changed gene sets, associated with biological processes, are organized by z-score.

**Figure 8:**
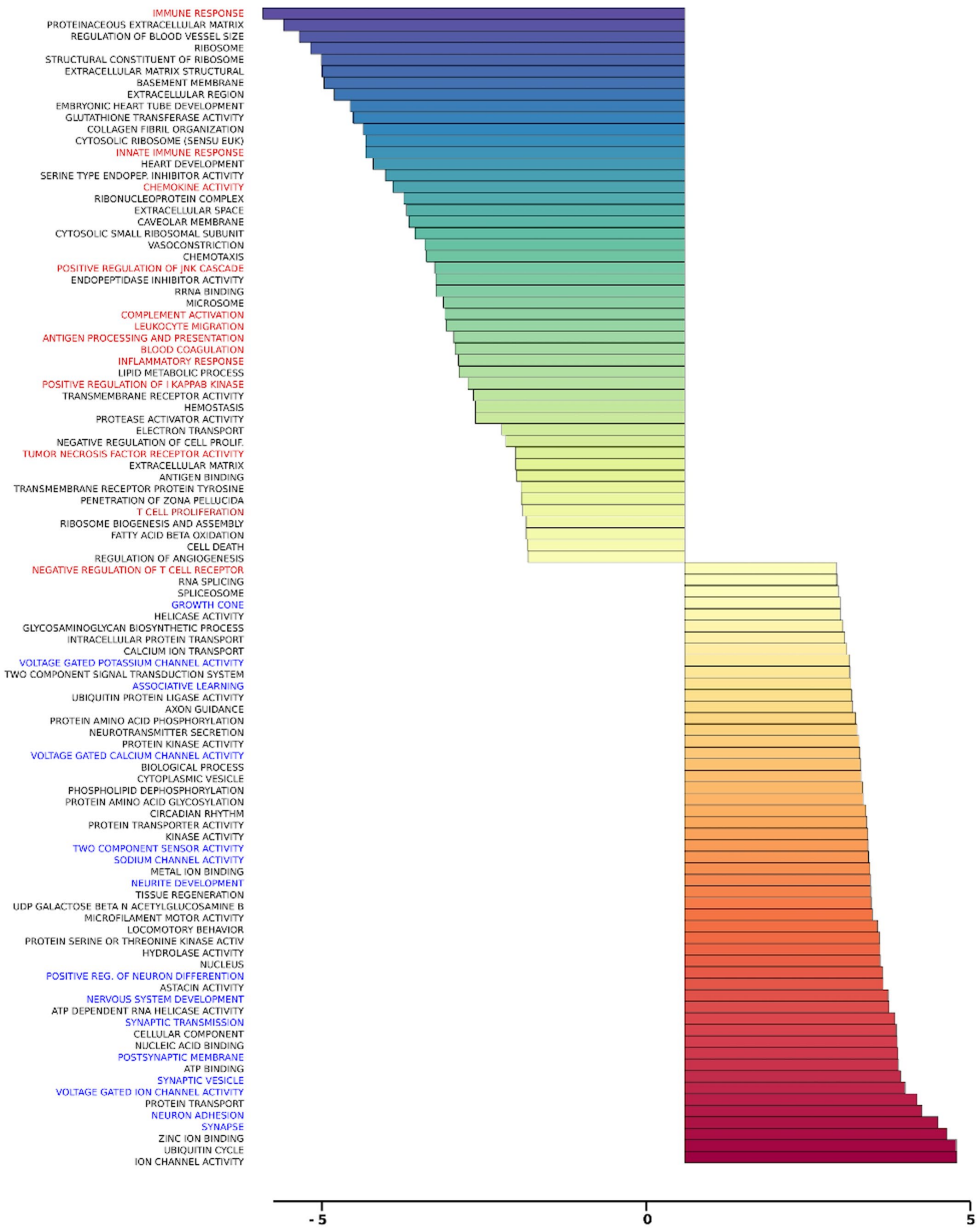
Gene ontology gene set analysis in the *in vivo* model, cortex, young. Highlighted in red text are gene groups involved in inflammation and immune response, and blue text highlights gene groups involved in synaptic growth and synaptic transmission in young rat cortical samples following iTBS compared to sham treatment. Significantly changed gene sets, associated with biological processes, are organized by z-score.

**Figure 9:**
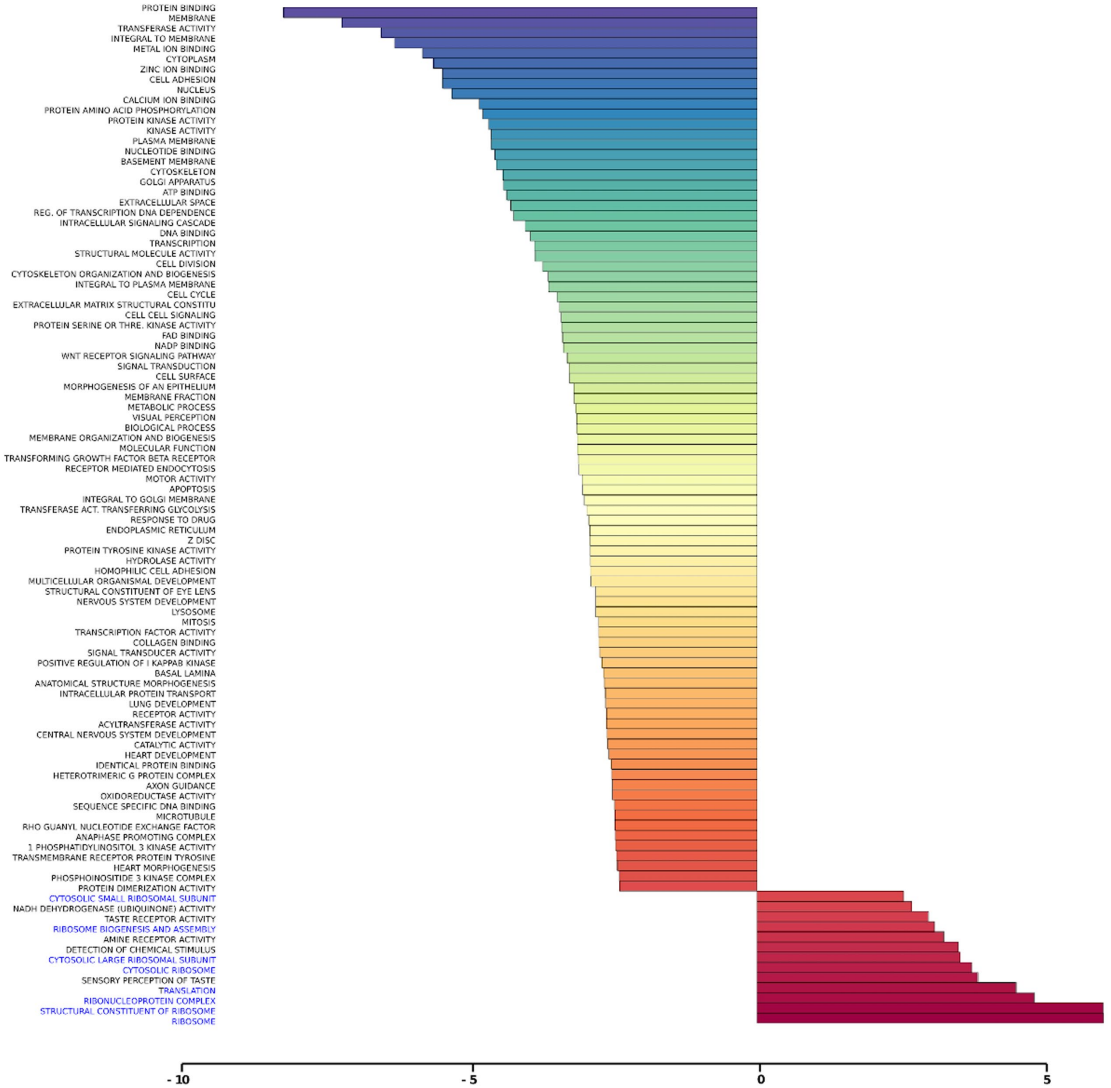
Gene ontology gene set analysis in the *in vivo* model, hippocampus, young. Highlighted in blue are gene groups involved in ribosomal function of young hippocampus samples following iTBS compared to sham treatment. Significantly changed gene sets, associated with biological processes, are organized by z-score.

Gene set analysis is based on gene expression changes in functionally related groups of genes, as opposed to individual genes, resulting in statistically significant aggregate scores for each gene group. Figure 6 displays a marked reduction in Gene Ontology (GO) inflammatory gene sets in the *ex vivo* young hippocampus using 1 Hz stimulation versus the unstimulated sham. These include reductions in Innate Immune Response, Complement pathways, as well as Chemokine and Cytokine GO gene sets. Broad-based immune suppression was found in the *ex vivo* young hippocampus model using iTBS versus sham as well, including gene sets for Inflammatory Response, Chemotaxis, Antigen Processing and Presentation, Immunoglobulin Mediated Immune Response, among others (Figure 7). In addition to marked immune suppression, upregulated GO gene sets in the iTBS *ex vivo* model included a theme of mitochondrial and energy-related GO gene groups. Immune suppression is evident in the *in vivo* model in the cortex after iTBS stimulation: GO gene sets such as Immune Response, Complement Activation, and Chemokine activity, among others, were downregulated, and upregulated gene sets included Synaptic Vesicle, Synaptic Transmission, and Neurite Development (Figure 8). In the *in vivo* model in the hippocampus, a theme of translation and ribosomal-related gene sets were upregulated after iTBS (Figure 9). For the *in vivo* iTBS model, alterations in GAD sets for inflammatory disorders such as Scleroderma, Crohn’s disease, and Lupus, were found in both cortex and hippocampus, as well as disorders specifically relevant to TMS treatment including mood disorders, seasonal affective disorders, attention-deficit/hyperactivity disorder, schizophrenia, substance use related disorders, among others (Supplementary Figures 2–5).

## Discussion

The last decade has witnessed a significant increase in pre-clinical and clinical research on rTMS as a therapeutic tool to treat several neuropsychiatric conditions. However, the molecular basis of rTMS effects remains largely unexplored. Here, using three experimental rat models, we show that the transcriptional response to rTMS treatment is complex and dynamic. Widespread transcriptional responses were apparent in the neuronal *in vitro* culture model, in the *ex vivo* hippocampal slice model, and in different brain regions in the *in vivo* model. Our results have implications for both research and clinical settings focused on the use of rTMS as a treatment for neurological conditions.

### Alterations in glutamatergic and GABAergic signaling pathways following rTMS are specifically relevant to neuropsychiatric disorders

Using rat hippocampal neuronal cultures, we observed that rTMS regulated the expression of genes involved in inhibitory and excitatory neurotransmission mediated in part by glutamatergic and GABAergic synapses. The evidence supporting this argument can be found in Table 2 and Figures 3A,B. In Table 2, we present results highlighting the specific genes that exhibit alterations following rTMS. These findings demonstrate that rTMS had a notable impact on the expression of various glutamatergic and GABAergic genes. In addition, Figures 3A,B depict the changes in gene expression induced by rTMS. These figures illustrate the specific genes in the glutamatergic and GABAergic pathways that are significantly modulated by rTMS. The observed alterations support the suggestion that rTMS is positioned to potently influence inhibitory and excitatory neurotransmission.

Alterations in the glutamatergic system can promote excitotoxic cell death, comprising a potential mechanism of neurodegeneration in Alzheimer’s disease (Mufson et al., 2008; Wang and Reddy, 2017). Early aberrant excitatory neurotransmission is frequently observed in both animal models of Alzheimer’s disease (Palop et al., 2007) and patients (Scott et al., 2002), and blocking the action of glutamate and targeting excitatory synapses has been exploited as a potential pharmacological treatment for the disease. Altered oscillatory rhythmic activity and network hypersynchrony are also features of Alzheimer’s disease (Koch et al., 2022) and may contribute to cognitive impairment (Busche and Konnerth, 1700; Palop and Mucke, 2016). However, while a major emphasis in prior research has focused on dysfunction of the glutamatergic system, accumulating evidence links inhibitory GABAergic interneurons to excitatory/inhibitory imbalance as a potential early contributor to cognitive impairment in both aging (Oh et al., 2010; Gallagher et al., 2019) and disease (Li Y. et al., 2016; Govindpani et al., 2017).

Disruption in excitatory/inhibitory balance has also been associated with other neuropsychiatric and neurological conditions such as chronic stress (Varga et al., 2017), major depression disorder (Pehrson and Sanchez, 2015; Fee et al., 2017; Fogaca and Duman, 2019), autism spectrum disorder (Schur et al., 2016), bipolar disorder (Bialer, 2012), epilepsy (Ye and Kaszuba, 2017; Losi et al., 2019) and schizophrenia (Metzner et al., 2019), and normalizing altered inhibitory function represents a promising target for therapeutic intervention. rTMS has been identified as a modifier of GABAergic (Trippe et al., 2009; Jazmati et al., 2018) and glutamatergic (Yang et al., 2014) systems previously and preventing or reversing disease-related imbalance by targeting either system with rTMS could be of both symptomatic and disease-modifying value.

### Transcriptional changes related to immunosuppression are relevant to inflammatory effects in depression and Alzheimer’s disease

A major insight from our analysis is the broad transcriptional change related to immunosuppression, observed as early as 2 h after stimulation in hippocampal slices. Neuroinflammation has emerged as a key feature in the pathogenesis of Alzheimer’s disease, potentially playing a causative role rather than simply as a secondary consequence of the pathological cascade (Sheng et al., 2003). Given the failure of amyloidogenic drugs to provide therapeutic benefit, scientific interest has shifted to other features of neurodegeneration including neuroinflammation (Castello et al., 2014). Similarly, major depressive disorder has been associated with increased activation of the immune system (Lee and Giuliani, 2019), and many front-line pharmaceutical treatments for depression have been shown to reduce inflammatory activation and lower circulating cytokine levels (Galecki et al., 2018).

rTMS has proven effective in animal models as a therapeutic tool targeting the inflammatory response (Sasso et al., 2016). Our results are consistent with a potential anti-inflammatory benefit, demonstrating that stimulation-induced downregulation of genes related to immunosuppression in young hippocampal slices 48 h following treatment. Strikingly, however, while decreased neuroinflammatory gene transcription was the predominant effect in young, rTMS had the opposite effect in hippocampal slices from aged animals with cognitive impairment (AI), inducing a potential proinflammatory transcriptional response. Given that aging itself is associated with chronic increases in circulating levels of inflammatory markers (Singh and Newman, 2011) – a phenomenon exacerbated by age-associated diseases (Chung et al., 2009) – our results suggest that rTMS might exacerbate age-related pathological immune activity. Thus, rTMS treatments with demonstrated anti-inflammatory benefits in young adults may have unanticipated effects in older recipients. Future studies focusing on the neuroinflammatory effects of rTMS specifically in the context of aging will be needed to move this technology forward in the clinical setting.

Although the mechanisms that mediate neuroinflammation are not fully understood, it is well-accepted that microglia play a key role (DiSabato et al., 2016; Dokalis and Prinz, 2019). In our study, the comparative analysis of purified hippocampal neuronal culture preparations versus the *ex vivo* or *in vivo* complex tissue samples, where glial cells are present, suggests that the observed influence on inflammatory response genes likely arises from the non-neuronal compartment. Taking advantage of the unique dataset generated in these experiments, future analyses directly comparing the transcriptional response to rTMS as a function of experimental preparation, temporal kinetics, age, and cognitive status will provide a rich source of insight into the complex molecular consequences underlying the phenotypic response to intervention.

### Our *ex vivo* experiments provide an approach to systematically test rTMS-drug interactions

Although FDA-approved as a stand-alone treatment for non-responsive major depressive disorder, in clinical practice rTMS is typically used as a supplementary therapy, together with psychotropic medication. A therapeutic benefit of combined rTMS and adjunctive drug treatment for major depression has been confirmed previously (Wei et al., 2017), but evidence fully exploring the interaction between rTMS and anti-depressants is scarce. Psychotropic drugs affect cortical excitability and plasticity (Minzenberg and Leuchter, 2019) and medications commonly used to treat neurological conditions change neural circuit and network activation (Borchert et al., 2016; Zheng et al., 2017). The use of concomitant medication may impact rTMS treatment outcome favorably or unfavorably, depending on the drug category or mechanism of action (Hunter et al., 2019). For example, while still in their infancy, studies in pharmaco-TMS have shown that medications that block voltage-gated sodium channels increase the evoked motor threshold to TMS, i.e., a common metric used to normalize and titrate stimulation intensity across individuals (a readout for TMS dosage; Ziemann, 2004; Ziemann et al., 2015).

The idea that the effects of rTMS vary depending on the use of concurrent medication aligns with the ‘state-dependency’ concept of TMS (Silvanto and Pascual-Leone, 2008). Among the factors that might influence the outcomes of TMS, the pharmacological ‘state’ of the brain has received relatively limited attention, likely because patients treated with rTMS for neuropsychiatric conditions are typically receiving concurrent pharmacological treatment. In this context, our *ex vivo* experiments point to one potentially useful approach for research aimed at identifying drug-rTMS interactions. Overall, while recognizing that the barriers to successful translation are substantial, properly designed basic research can nonetheless inform therapeutic development toward safer and more effective TMS application in a variety of conditions.

### The complex transcriptional profiles induced by rTMS in the context of age, tissue, and cognitive status are relevant to the clinical status of individual patients

It has been over a decade since the first rTMS application was approved by the FDA for the treatment of major depressive disorder, and since then the range of potential applications under investigation with rTMS has skyrocketed. The research on rTMS as a therapy includes patients with a variety of disorders and pathological signatures, from all ages and different cognitive statuses. Recent *in vivo* data points to the potential translational relevance of findings in animal models demonstrating effects on behavior and structural plasticity (Cambiaghi et al., 2022) and the potential implication for clinical application in neurodegenerative diseases (Weiler et al., 2020). In our results, we observed that rTMS induced unique gene expression profiles in each experiment, all of which reflected a variety of biological conditions and different stimulation parameters. In addition, the gene expression response following rTMS was highly complex. Whereas previous studies investigating the effects of rTMS have focused on alterations in only a handful of *priori*-selected genes (Lee et al., 2014; Wang et al., 2014; Grehl et al., 2015; Cirillo et al., 2017; Legrand et al., 2018; Cui et al., 2019; Wu et al., 2022), our large-scale analysis of gene expression detected several complex different pathways that are altered after stimulation.

Any non-invasive brain stimulation protocol intended to target a specific gene set or mechanism in the context of treating a given condition may have unexpected consequences in a different context, e.g., in other brain regions, or as a function of the cognitive status and age of the recipients. For example, clinical trials of rTMS (Turriziani et al., 2012) have reported that the same stimulation protocol that yields cognitive improvement in some subjects at risk for Alzheimer’s disease has detrimental effects in cognitively healthy individuals. Likewise, stimulation aiming at targeting a specific pathway or mechanism may induce changes in other genes and unintended downstream pathways. The findings reported here also highlight that the transcriptional response can be temporally dynamic, resulting in both increases and decreases in the same gene families across time, suggesting that a protocol chosen for its acute effects may lead to unpredictable long-term changes in expression.

Taken together, our results suggest that patterns of gene expression following rTMS are complex, dynamic, and dependent on the brain region, age, cognitive status, and potentially many other subject variables not examined here. Given the current and growing clinical application of rTMS, it is timely that greater attention turns to basic research aimed at understanding the underlying basis of reported therapeutic benefits, with the goal of optimizing treatment in the context of the individual patient.

The limitations of research on non-invasive brain stimulation in experimental animal models are significant. A primary limitation from a translational perspective is that, although we analyzed multiple stimulation protocols, we only studied the effects of one stimulation session, while clinical rTMS approaches employ treatments over the course of days or weeks. Further studies investigating the effects of rTMS on a long-term basis should be conducted. Another limitation is that although in some cases we used a diverse parametric setting such as different ages and cognitive states, our initial report is predominantly focused on the effects of rTMS itself. Here, each rTMS-stimulated sample group was statistically compared to its own unstimulated sham control, as opposed to comparisons across age and between cognitive groups. Detailed results of the transcriptional changes due to rTMS treatment in the context of age, cognition, and brain region will be presented elsewhere.

## Data availability statement

The three data sets underlying this paper have been deposited to the NCBI Gene Expression Omnibus and are accessible through their individual GSE identifiers (in vitro: GSE230147, (ex vivo: GSE230148, and in vivo: GSE230149) and the GEO SuperSeries accession number GSE230150 (https://www.ncbi.nlm.nih.gov/geo/query/acc.cgi?acc=GSE230150).

## Ethics statement

The animal study was approved by Animal Care and Use Committee of the Intramural Research Program of the NIA. The study was conducted in accordance with the local legislation and institutional requirements.

## Author contributions

MW, KCS, EL, JML, KGB, and PRR: study conception and design. MW, KCS, EL, SC, JML, MPM, KGB, and PRR: data collection. MW, KCS, KS, JPK, WHW, YZ, PC, EL, JML, KGB, and PRR: analysis and interpretation of results. MW, EL, JML, KGB, and PRR: draft manuscript preparation. All authors contributed to the article and approved the submitted version.
