# Supplementary figures and images for: Transcriptional changes in the rat brain induced by repetitive transcranial magnetic stimulation
Source: Front Hum Neurosci. 2023 Nov 13;17:1215291. doi: 10.3389/fnhum.2023.1215291 (PMC10679736; doi:10.3389/fnhum.2023.1215291)

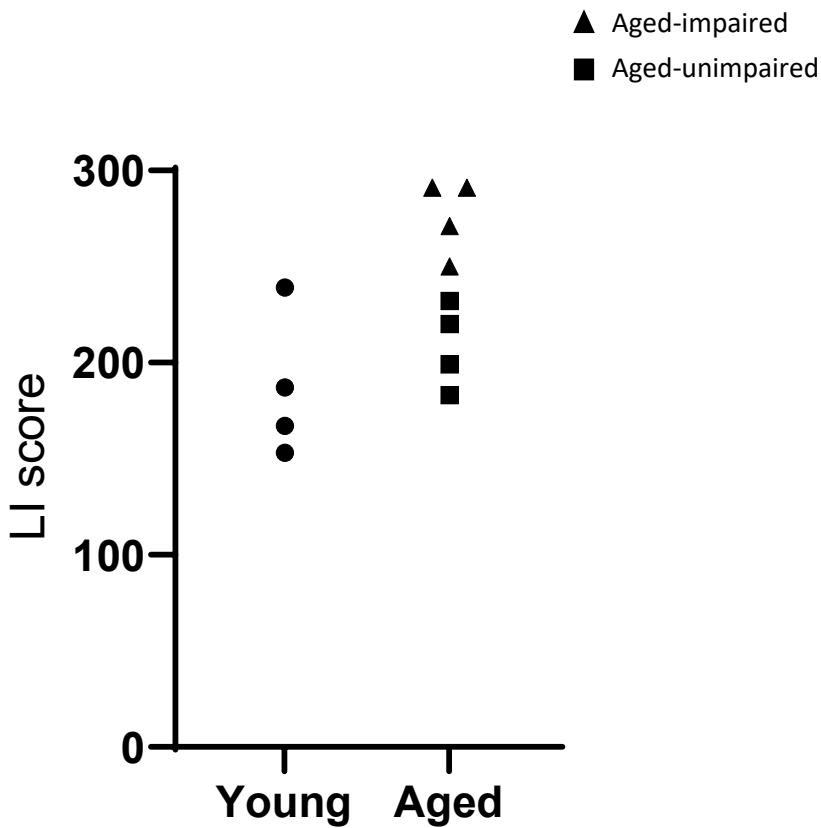

Supplement: Supplementary file 1 [file Image_1.pdf]

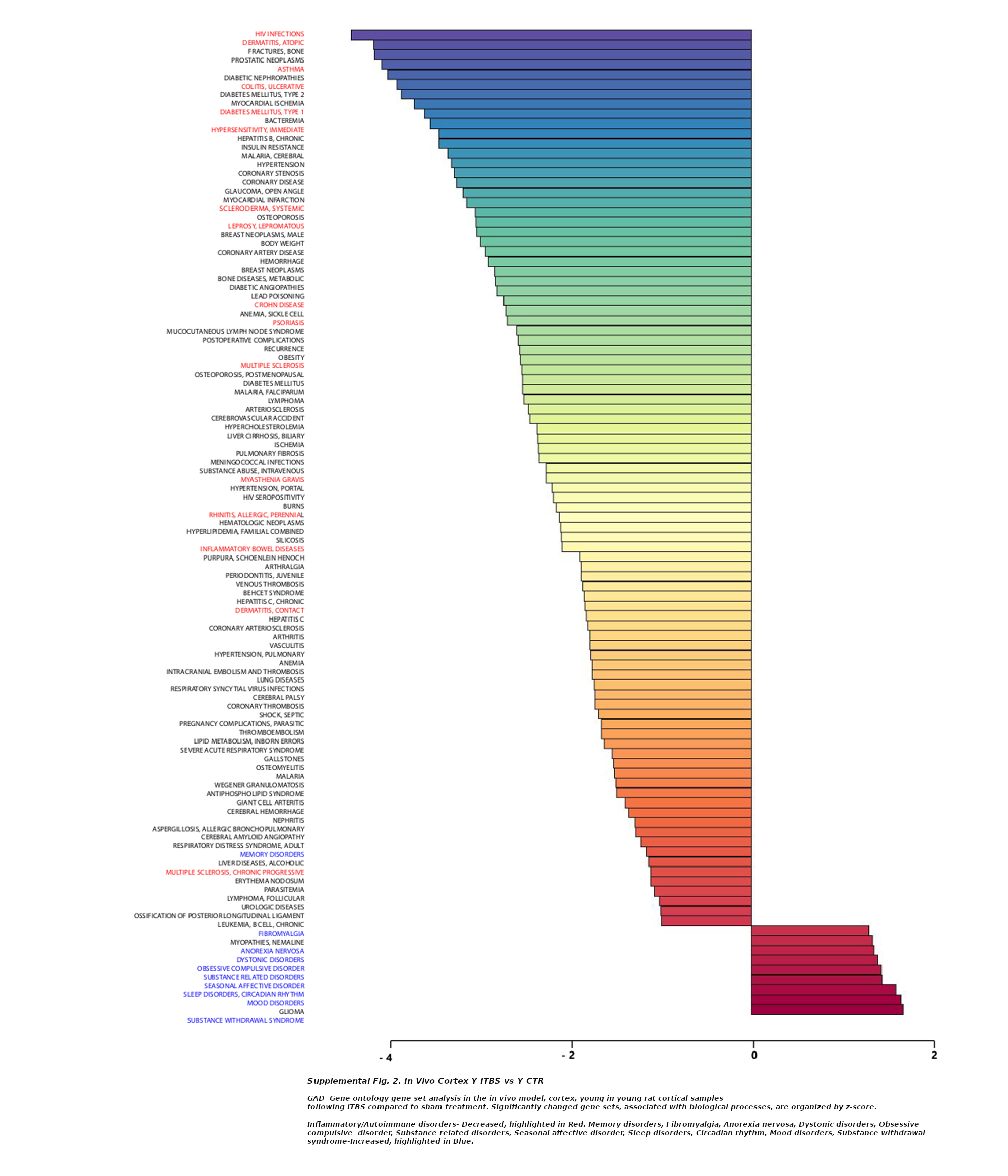

Supplement: Supplementary file 2 [file Image_2.tif]

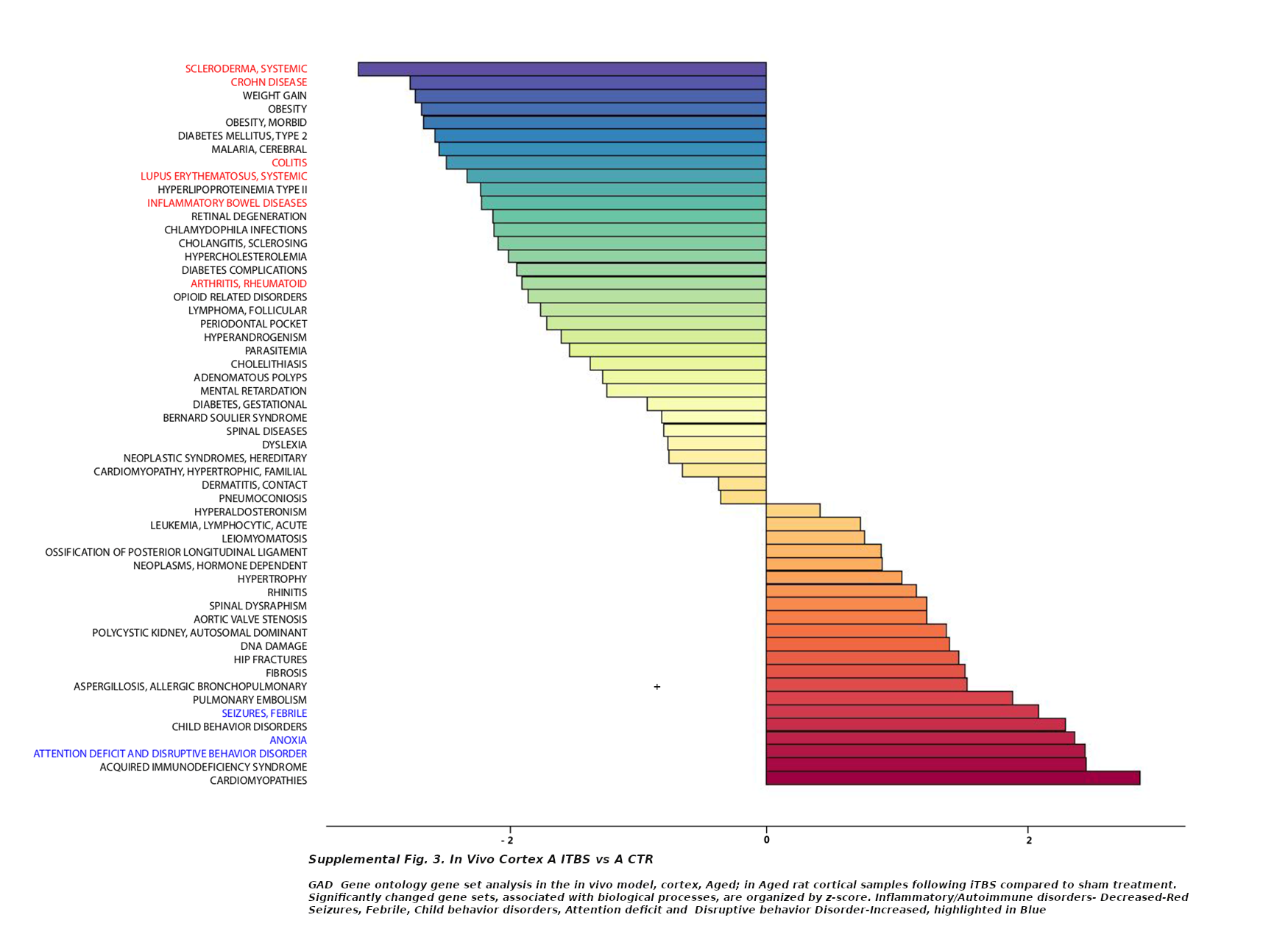

Supplement: Supplementary file 3 [file Image_3.tif]

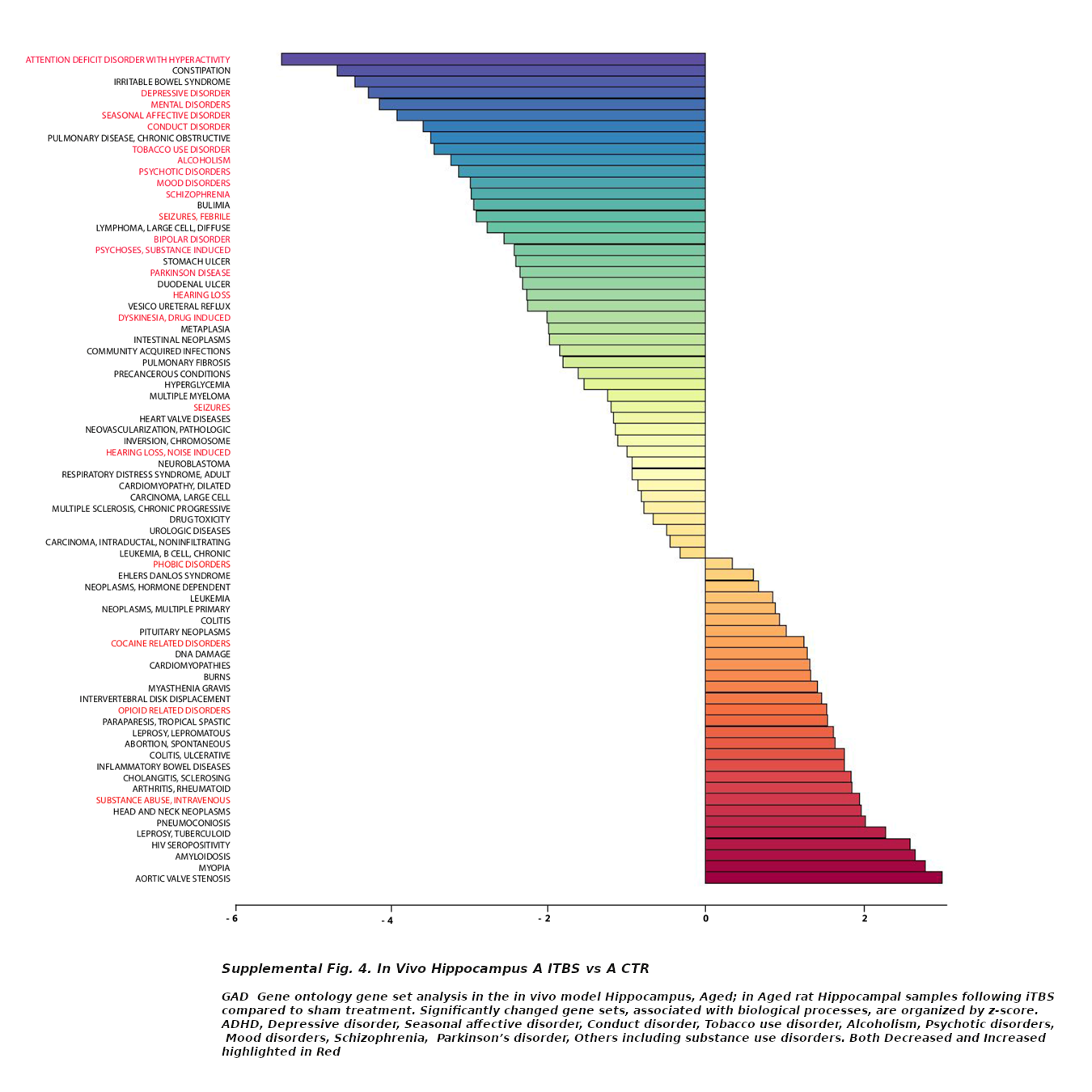

Supplement: Supplementary file 4 [file Image_4.tif]

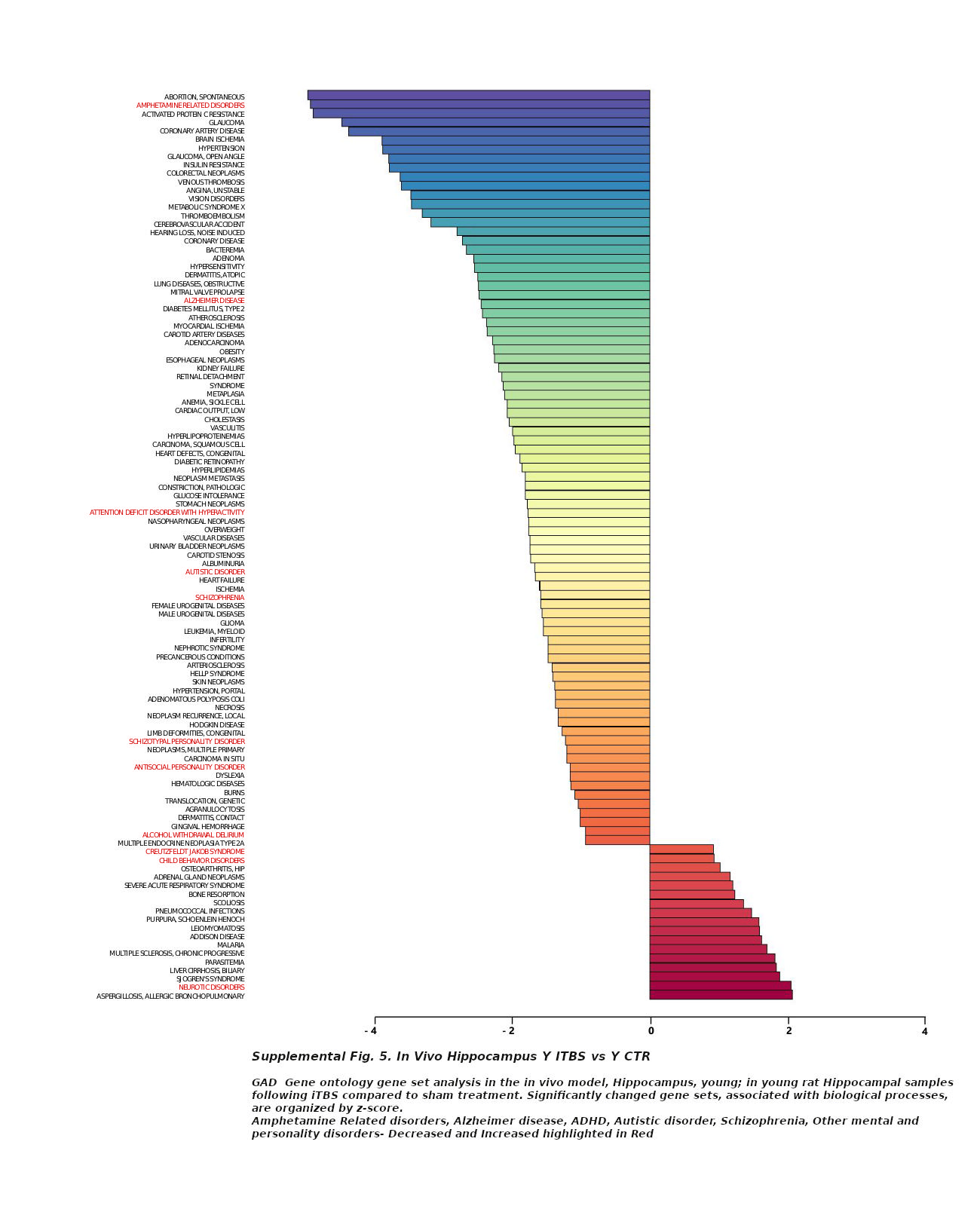

Supplement: Supplementary file 5 [file Image_5.tiff]
